# Bioplastic Production Using Natural Extracts with Cellulose Assisted by Experimental and Computational Screening

**DOI:** 10.3390/molecules30132752

**Published:** 2025-06-26

**Authors:** Lizbeth Zamora-Mendoza, Jhonny Caicho, José R. Mora, Daniela Negrete-Bolagay, Victor H. Guerrero, Noroska G. S. Mogollón, Melanie Ochoa-Ocampo, Jefferson Pastuña-Fasso, José F. Álvarez Barreto, Sebastián Ponce, Juan Paredes, Henry Erazo, Patricia I. Pontón, Marco León, Frank Alexis

**Affiliations:** 1 Departamento de Ingeniería Química, Colegio de Ciencias e Ingenierías, Universidad San Francisco de Quito (USFQ), Quito 170901, Ecuador; lizbethzamoramen@gmail.com (L.Z.-M.); jhonnycaicho1@gmail.com (J.C.); jrmora@usfq.edu.ec (J.R.M.); jalvarezb@usfq.edu.ec (J.F.Á.B.); sponce@usfq.edu.ec (S.P.); 2 Departamento de Materiales, Escuela Politécnica Nacional (EPN), Quito 170143, Ecuador; daniela.negrete@epn.edu.ec (D.N.-B.); victor.guerrero@epn.edu.ec (V.H.G.); patricia.ponton@epn.edu.ec (P.I.P.); 3 Biomolecules Discovery Group, Universidad Regional Amazónica Ikiam, Tena 150101, Ecuador; noroska.salazar@ikiam.edu.ec; 4 Laboratorio de Productos Naturales, Universidad Regional Amazónica Ikiam, Km 7 Vía Muyuna, Tena 1701518, Ecuador; melanie.ochoa@ikiam.edu.ec (M.O.-O.); jefferson.pastuna@ikiam.edu.ec (J.P.-F.); 5 Facultad de Ingeniería Civil y Mecánica, Universidad Técnica de Ambato (UTA), Ambato 180207, Ecuador; jgparedes@uta.edu.ec (J.P.); henryerazo1995@gmail.com (H.E.); 6 Institute for Energy and Materials, Colegio de Ciencias e Ingenierías, Universidad San Francisco de Quito (USFQ), Quito 170901, Ecuador; mleond@usfq.edu.ec; 7 Mechanical Engineering Department, Colegio de Ciencias e Igenierías, Universidad San Francisco de Quito (USFQ), Quito 170901, Ecuador

**Keywords:** bioplastic, cellulose, natural dissolvent, plant extract

## Abstract

The increasing demand for sustainable and environmentally friendly materials has prompted intensive research into developing bioplastics as viable alternatives to conventional petroleum-derived plastics. Here, we report a novel approach to bioplastic production by employing plant extract-based solvents to partially dissolve cellulose, a fundamental biopolymer precursor. Using plant-derived solvents addresses concerns surrounding the environmental impact of traditional solvent-based processes, as per the principles of green chemistry. Using computational screening, some natural products were identified from the integrated database resource MEGx. Six natural sources were selected based on their molecular weight, high pKa, and chemical classification. Thin-layer chromatography (TLC) and column chromatography confirmed the presence of molecules in the extracts. Bioplastics were prepared with 1, 3, 6, 10, and 15 wt.% plant extract concentrations. Control samples without conventional dissolved and positive controls were also studied to compare their properties with novel bioplastics. Chemical characterization and biodegradability tests were performed. Degradation in water and soil tests for 35 days showed that the biodegradability of the bioplastics with natural extracts at higher concentrations was faster than that of the control samples. By day 35, bioplastics containing 15 wt.% of the D1 W extract showed rapid degradation, with higher weight loss compared with the conventional controls. The positive control (C4), containing NaOH and glycerol, degraded more slowly than the plant extract-based formulations. Also, the test indicated that the natural dissolvent’s influence on the water uptake of the material produced a better performance than the control samples. The surfaces of the bioplastic formulations were analyzed using a scanning electron microscope (SEM) at different magnifications. The findings presented here hold promise for advancing the field of bioplastics and contributing to the sustainable utilization of plant resources for eco-friendly material production.

## 1. Introduction

**Figure 1:**
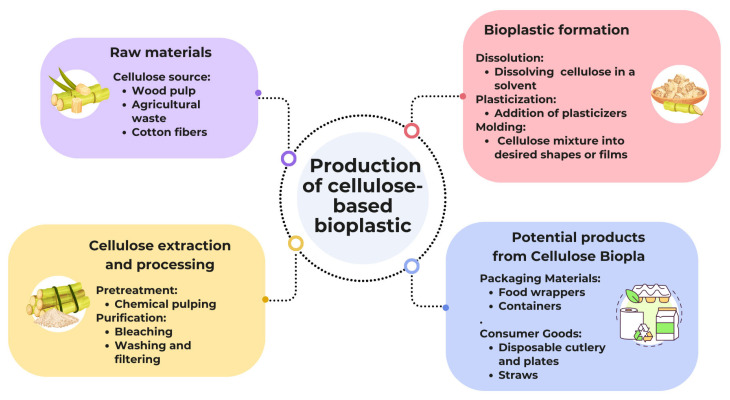
Production of cellulose-based bioplastic and its potential products.

Since the 1950s, around 8.3 billion tons of fossil fuel-based plastic have been generated, and it should be highlighted that 80% of it is not recyclable, affecting the environment [1]. Plastic waste generates byproducts associated with phthalates and bisphenols in groundwater and soil contamination [2]. The global environmental crisis, driven by the ubiquitous presence of petroleum-based plastics in our ecosystems, has spurred a dire need for sustainable alternatives [3]. Bioplastics are commercial polymer products obtained from natural sources [4]. Bioplastics have emerged as a promising solution due to the biodegradability of plastic and its reduced environmental impact. Among the various bioplastic precursors, cellulose (Figure 1), a ubiquitous biopolymer abundant in plants, holds great potential [5]. Cellulose is a polymer of repeating glucose units linked by glycosidic bonds [6]. However, the dissolution of cellulose into a workable form for bioplastic production remains a challenge [7].

Conventionally, sodium hydroxide action on glycosidic linkages typically results in depolymerization because glycosidic bonds are the primary covalent linkages connecting glucose units in cellulose chains [8]. When this solvent is introduced, it weakens the interactions between hydroxyl groups within and between cellulose molecules, reducing the crystalline order and promoting swelling and partial dissolution [9]. In the viscose process, the alkali cellulose is derivatized into cellulose xanthate through a reaction with carbon disulfide (CS_2_) [10]. This step is crucial for creating a cellulose solution that can be used to produce bioplastics. An alkaline solution of sodium could partially dissolve 13% of the biopolymer from cellulose [11].

Glycerol is used primarily as a plasticizer in the formulation of cellulose-based bioplastics to improve flexibility and prevent brittleness [12]. Glycerol is a naturally occurring substance that is biodegradable, contributing to the overall sustainability of bioplastics [13]. It is safe for various applications, including food packaging and medical products, making it suitable for use where human exposure is possible [13]. As a byproduct of biodiesel production, glycerol is often less expensive than traditional petroleum-based plasticizers, reducing production costs for bioplastics [14]. It is particularly effective with hydrophilic materials, such as starch, enhancing the mechanical properties of bioplastic formulations [15]. Therefore, glycerol’s combination of sustainability, safety, and performance makes it a valuable bioplasticizer in the evolving landscape of eco-friendly materials. Cellulose, though abundant and biodegradable, tends to form rigid structures when processed alone, limiting its usability in plastic applications [16]. Glycerol reduces the intermolecular forces between cellulose chains, enhancing their mobility and making the material more pliable [17]. Its hygroscopic nature also helps retain moisture, preventing the bioplastic from becoming brittle over time [18].

Traditional methods of cellulose dissolution involve using harsh chemicals such as NaOH and solvents, often contributing to pollution and environmental risks [19]. Plant extract-based solutions have gained considerable attention in the quest for greener and more sustainable approaches. These solvents, derived from renewable plant sources such as agricultural residues, offer the promise of cellulose dissolution with reduced ecological harm. Recent research has discussed the role of hydrophobic interactions and H-bonding in the dissolution of cellulose [20]. New studies have the main goal of developing a new aqueous-based solvent system for cellulose’s partial or total dissolution [21].

**Figure 2:**
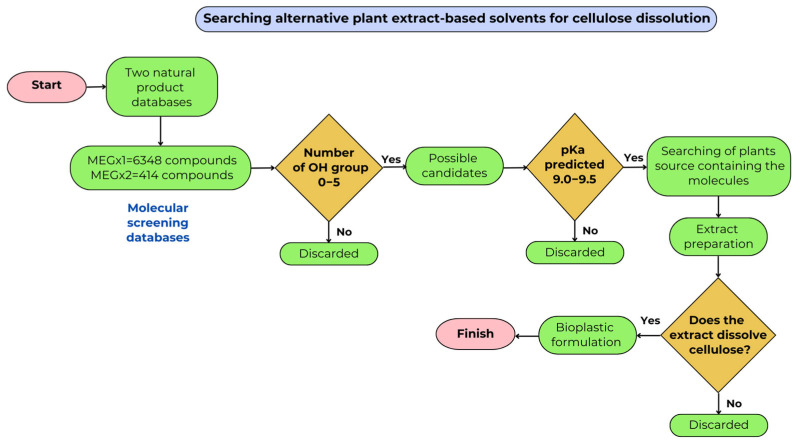
Diagram for searching for alternative plant extract-based solvents for partial dissolution of cellulose used in bioplastic formulation.

This research aimed to study cellulose solvents derived from plant extracts, with a focus on generating bioplastics. Hemp has a higher cellulose content and yields more cellulose than other wood sources [22]. A hemp cellulose-based polymer was mixed with plant extract-based solvents and a plasticizer to form a bioplastic material with the desired properties. By harnessing the use of computational screening to select potential molecules with high pKa, hydroxyl groups for high solubility in water, and no toxicity, which has never been explored for the production of bioplastics, this research aimed to contribute to the bioplastics industry using an environmentally responsible approach (Figure 2) [23].

Our research tested six plant extract-based dissolvents derived from phenylalanine (quinoa, D1 W), abrine (guayaba, D2 W), alpha-Boswellic acid acetate (palo santo, D3 W), bellericagenin B (Ivory Coast almond, D4 W), obtusol (cacalosuchil, D5 W), and 1-linoleoyl-sn-glycerol-3-phosphorylcholine (soya, D6 W) were used for cellulose bioplastic, focusing on their physical, molecular, and chemical properties. The outcomes of this study not only contribute to the advancement of bioplastic technology but also align with the global drive toward sustainable material production, offering a glimpse into a more eco-conscious future for the plastics industry.

## 2. Results

### 2.1. Chemical Characterization of Plant Extracts

#### 2.1.1. FTIR

**Figure 3:**
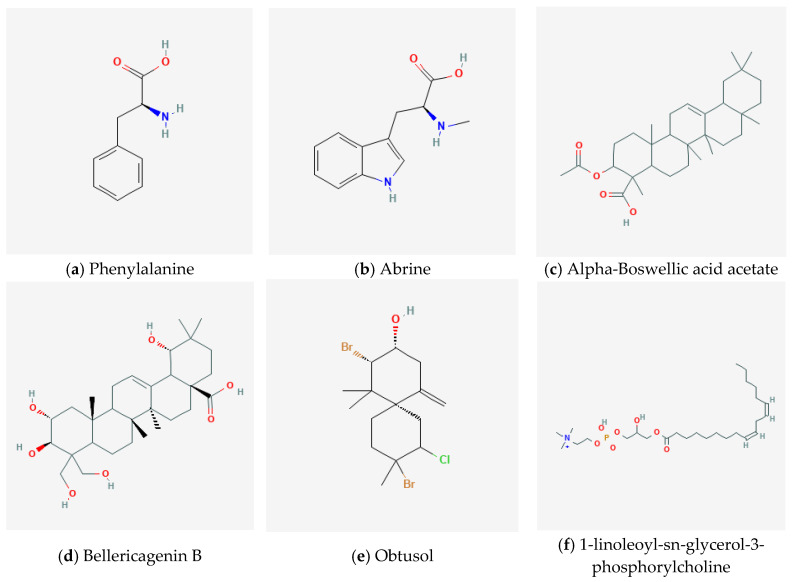
Chemical structures of target molecules obtained from plant extracts: phenylalanine (quinoa, D1 W), abrine (guayaba, D2 W), alpha-Boswellic acid acetate (palo santo, D3 W), bellericagenin B (Ivory Coast almond, D4 W), obtusol (cacalosuchil, D5 W), and 1-linoleoyl-sn-glycerol-3-phosphorylcholine (soya, D6 W). Information obtained from https://pubchem.ncbi.nlm.nih.gov/ (accessed on 2 June 2025).

After extracting the natural sources of our six chosen molecules (see Section 3), we performed IR of these extracts to determine whether the desired products were present. The chemical structures of the target molecules are presented in Figure 3.

**Figure 4:**
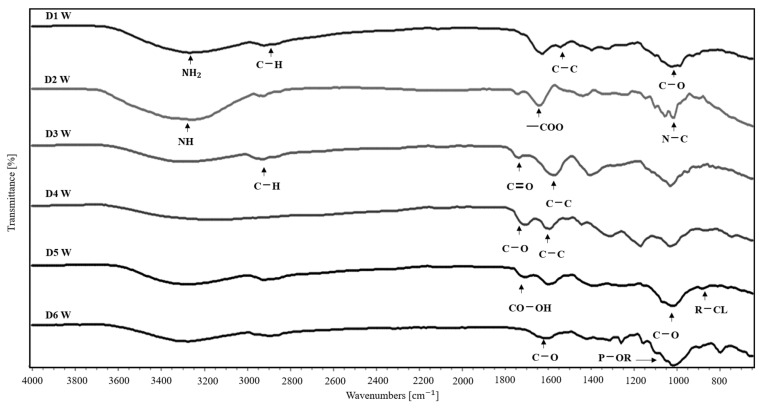
FTIR spectra of extracts: phenylalanine (quinoa, D1 W), abrine (guayaba, D2 W), alpha-Boswellic acid acetate (palo santo, D3 W), bellericagenin B (Ivory Coast almond, D4 W), obtusol (cacalosuchil, D5 W), and 1-linoleoyl-sn-glycerol-3-phosphorylcholine (soya, D6 W).

The absorption spectra of the dried extracts obtained in the range of 4000 and 650 cm^−1^ are shown in Figure 4. The FTIR of the D1 W molecule shows the characteristic peaks at 3386 cm^−1^ that correspond to NH_2_, 3282 cm^−1^ is the –OH group, the peak at 2922 cm^−1^ shows C-H stretching of the alkyl group, there is a characteristic peak of the ring aromatic at 1999 cm^−1^, the peak at 1632 cm^−1^ corresponds to C=O, and C-C stretching is shown in the peak at 1399 cm^−1^, C-O stretching in the peak at 1328 cm^−1^, and the C-H group in the peak at 1040 cm^−1^, determining the presence of our molecule in the extract. The D2 W molecule has the characteristic peaks at 3441 cm^−1^ corresponding to the –OH group, the distinctive peak of the aromatic ring in 1993 and 1913 cm^−1^, and a peak at 1718 cm^−1^ corresponding to C=O stretching as well. Like N-H, the peak at 1590 cm^−1^ shows the presence of the -COO group, C-C stretching is ascribed to the peak at 1398 cm^−1^, and the C-O group corresponds to the peak at 1237 cm^−1^, characteristic of the molecules. The peak at 1028 cm^−1^ is shown for N-C stretching. Finally, the peaks at 817 cm^−1^ and 770 cm^−1^ correspond to C-H bending, showing the molecule could be present.

The D3 W molecule shows the characteristic peaks at 2930 cm^−1^ corresponding to the –CH group, the peak of the aromatic ring at 1994 cm^−1^, and C=O stretching at 1744 cm^−1^, and the peak at 1579 cm^−1^ confirms the presence of the C-C stretching of the aromatic ring, as well as the C-H group in the 1405 cm^−1^ peak, characteristic of the molecule. The 1030 cm^−1^ peak is shown for the cyclohexane structure, determining the presence of alpha-Boswellic acid acetate in the extract. The FTIR of the D4 W molecule shows the peak at 3433 cm^−1^ of the –OH group, the peak at 2927 cm^−1^ for –CH, and the peak at 1712 cm^−1^ corresponding to C=O. The 1602 cm^−1^ peak shows the presence of the C-C group and the C-O group at 1318 cm^−1^, characteristic of the molecule. The 1174 cm^−1^ peak is shown for the COO- structure, corresponding to the chemical molecule’s structure and the functional groups of bellericagenin B.

Furthermore, the D5 W molecule shows the characteristic peak at 3380 cm^−1^ corresponding to the –OH group, the distinct peak at 2922 cm^−1^ concerning –CH, the 1713 cm^−1^ peak corresponding to CO-OH, the 1600 cm^−1^ peak belonging to the C-C group of the aromatic group, and the C-O stretch group in the 1023 cm^−1^ peak, characteristic of the molecule. The 764 cm^−1^ peak shows the structure of C-Cl, and the 651 cm^−1^ peak that corresponds to C-Br is demonstrated. Therefore, the functional groups of the molecule of interest are shown. The FTIR of the D6 W molecule presented a peak at 3418 cm^−1^ corresponding to the –OH group, the characteristic peak at 2922 cm^−1^ belonging to –CH, and the peak at 2300 cm^−1^ corresponding to P-H stretching are also shown. The 1639 cm^−1^ peak shows the presence of the N-H group, as well as the C-O group in the 1584 cm^−1^ peak, characteristic of the molecule. The 1038 cm^−1^ peak shows the structure of P-OR and CH_2_.

#### 2.1.2. Phytochemical Screening

The samples were prepared at a concentration standard of 100 mg/mL, and the results of the preparation reagents are presented in Table 1. Phytochemical screening confirmed the presence of phytoconstituents like alkaloids, flavonoids, glycosides, phenols, lignins, saponins, sterols, tannins, anthraquinone, and reducing sugar [3]. To identify certain molecules of interest, a phytochemical study of the extracts was carried out to determine the presence or absence of the main groups of metabolites [24]. The extracts in aqueous solutions were assessed in a phytochemical analysis.

#### 2.1.3. Thin-Layer Chromatography Analysis

TLC is a widely used analytical tool because of its simplicity, relatively low cost, high sensitivity, and separation speed [25]. The TLC results are presented in Table 2. The TLC results of molecule D1 W were performed with the extract and the standard of the molecule (phenylalanine). The mobile phase corresponded to a butanol/acetic acid/water mixture, as described in [26], with the proportion adjusted to match the polarity of the molecule. This resulted in retention factors (Rf) of Rf_standard_ = 0.68 and Rf_D1 W_ = 0.65, demonstrating that phenylalanine was present in the extract. Similarly, the D2W extract was subjected to analysis and was found to be 0.59, while the Rf for the commercial abrine molecule standard was determined to be 0.67. This discrepancy suggests the absence of the abrine molecule in the D2 W extract, as evidenced by the ninhydrin 2% + ethanol revealing technique.

The TLC of the D3 W molecule was performed, and a retention factor of Rf_D3 W_ = 0.40 was obtained on the silica plate using toluene/ethyl acetate, with vanillin sulfuric acid as the developer for terpenoid molecules [27]. The D4 W molecule was performed using a mobile phase of hexane/acetone [28]. As a result, the retention factor of Rf_D4 W_ = 0.53 corresponded to the band of interest [29], whose identification was performed with the iodine developer and with UV light at 254 nm. In the case of D5 W, the Rf_D5 W_ = 0.38 could correspond to the molecule when the plate was exposed to UV light and the iodine chamber [30]. To confirm the presence of the molecule of interest, D6 W, the developer vanillin sulfuric acid ethanol was heated on the silica plate at 100 °C. The band corresponding to Rf_D6 W_ = 0.78 could confirm the presence of the molecule in the extract [31].

#### 2.1.4. Column Chromatography

**Figure 5:**
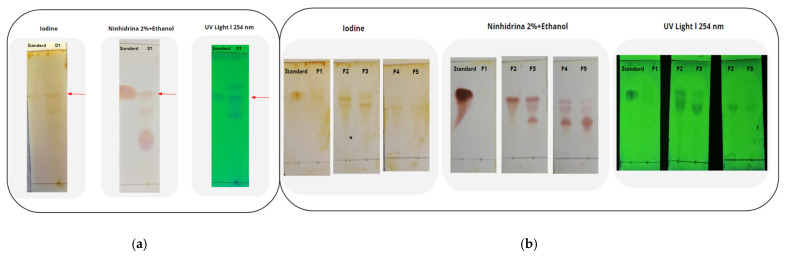
The complete extract D1 W presents the (**a**) composition and then the TLC of the five fractions (F1–F5) obtained from the column chromatography of the extract D1 W (**b**). It was identified that the F2 fraction contained the phenylalanine molecule (see red arrow), which is like the standard.

The column chromatography procedure employed for plant extracts represents a technique used for the isolation and identification of bioactive compounds within the extract under investigation [32]. Initially, 50 mL of the mobile phase, previously utilized in thin-layer chromatography (TLC), was prepared. The fully dried plant extract sample was subsequently combined with the mobile phase n-butanol/acetic acid/water to facilitate the distribution of the sample D1 W (see Figure 5). The solvent system was introduced into the column at consistent rates under gravity to fractionate D1 W. Each fraction was collected individually and sequentially numbered for subsequent analysis by thin-layer chromatography. Five fractions, each of 3.5 mL, were then stored for further examination. As a result, F2 showed the related molecule phenylalanine in the plant extract because the Rf_standard_ was 0.662, and the Rf_F2-D1 W_ was 0.657. Through this technique, the purity of the molecule in the plant extract was separated and identified.

#### 2.1.5. Metabolic Profile

The data were preprocessed in the MZmine software, version 3.9.0 (MZmine 3 Documentation), for non-targeted metabolomics of DDA-type data. Some of the parameters used in the software were a minimum MS1 peak height of 40 amplitude and an MS/MS abundance cutoff of 10 amplitude. Also, blank peaks present in the samples were removed. The results are presented in Table 3.

For the standardless identification of metabolites (level 2) the Global Natural Products Social Molecular Networking (GNPS) library (GNPS Documentation (ccms-ucsd.github.io) was used, resulting in .mgf or .mzML files that were converted from the Waters raw datasets according to the online instructions (Mass Spectrometry File Conversion—GNPS Documentation (ccms-ucsd.github.io). The mass tolerance of the precursor ion and that of the fragment ion were set to 0.02 Da. Subsequently, a molecular network was established in which edges were filtered to have more than three matching fragments and a cosine score greater than 0.70.

Level 1 identification: Level 1 metabolite identification of phenylalanine was carried out according to the minimum standards proposed for the metabolomics standards initiative (MSI), utilizing two criteria: (a) the alignment of the retention times with those of the authentic standard and (b) the match in *m*/*z* with a minimum of 10 ppm.

**Figure 6:**
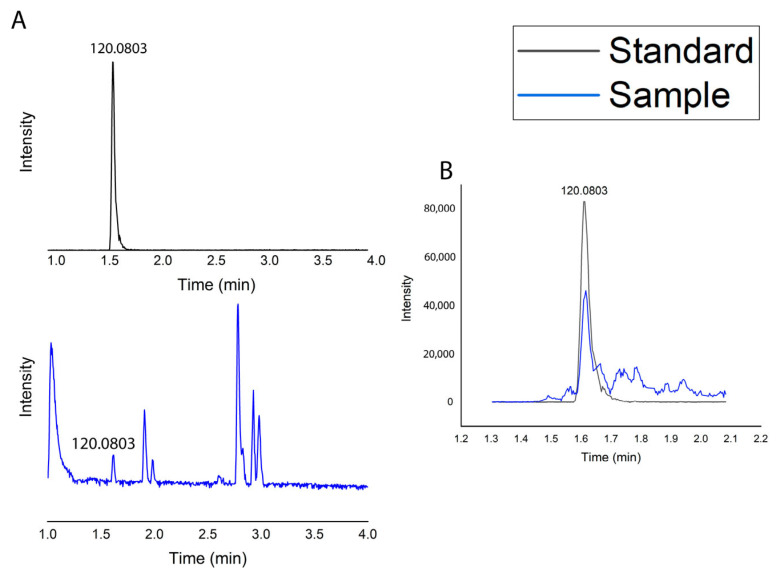
Chromatograms: (**A**) standard phenylalanine 1.615 min ion fragment (*m*/*z* 120.0806) and sample 1.615 min ion fragment (*m*/*z* 120.0803); (**B**) sample along with standard.

Level 2 metabolite identification: The raw profile MS/MS Waters files (*.raw) were mass-corrected in the MassLynx software V4.1 (see Figure 6). Each group of files (*.raw) was processed in MZmine 3.7.0 for 20 eV (ID = 9c3cd3dd39e44b8abba692c19ec8e17a) of collision energy to generate a list of feature height (*.csv) and spectra files (*.mgf) for level 2 metabolite identification using feature-based molecular networking (FBMN) algorithms on the Global Natural Products (GNPS) platform. Finally, the sample D1 W chromatogram showed a peak at 1.615 min, indicating that the compound in the sample had the same retention behavior in the chromatographic system as the standard phenylalanine [34]. Mass spectrometry provided the ion fragment *m*/*z* 120.0806 for the phenylalanine standard, corresponding to its molecular structure. The ion fragment for the sample was *m*/*z* 120.0803, which was almost identical to the standard. When the sample was analyzed alongside the standard (chromatogram B), the retention time and ion fragments remained consistent, further confirming the presence of phenylalanine in D1 W.

### 2.2. Preparation of Cellulose-Based Bioplastic Samples

**Figure 7:**
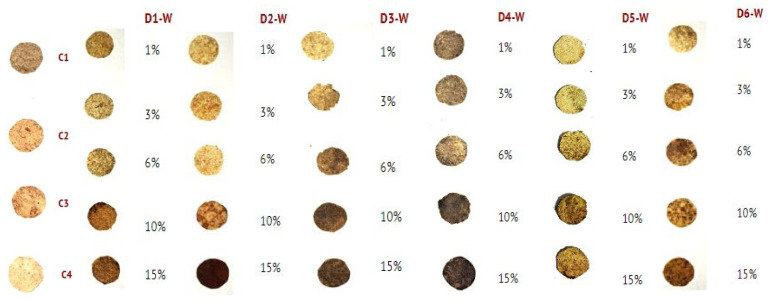
Bioplastic films using glycerol and a plant extract solvent concentration. Bioplastics with the plant extracts phenylalanine (quinoa, D1 W), abrine (guayaba, D2 W), alpha-Boswellic acid acetate (palo santo, D3 W), bellericagenin B (Ivory Coast almond, D4 W), obtusol (cacalosuchil, D5 W), and 1-linoleoyl-sn-glycerol-3-phosphorylcholine (soya, D6 W) compared with controls C1 (cellulose), C2 (cellulose + glycerol), C3 (cellulose + NaOH), and C4 (cellulose + NaOH + glycerol).

The cellulose was extracted from hemp with a yield of ±47.5% by mass. This extracted cellulose was then employed in the preparation of bioplastic materials. The preparation of the bioplastic commenced following the extraction and characterization of the six extracts. Cellulose, glycerol, and the plant extract were used as solvents in the bioplastic formulation. The concentration of the plant extract was varied to assess its effect. Each mixture was stirred magnetically for 60 min at 80 °C, followed by hot pressing at 240 °C to form bioplastic films. The films were subsequently cut into 1.791 cm diameter circular discs. Cellulose-based bioplastics were successfully produced using the extract-based natural solvent, except for the D2 W samples and the control C2, as illustrated in Figure 7. Notably, control sample C2 (without the plant extract) and sample D2W exhibited poor film-forming properties, resulting in brittle materials that were difficult to manipulate, unlike other formulations. This suggests that while cellulose dissolution occurred to some degree in all cases, the presence and type of plant extract significantly influenced the behavior of the final bioplastic.

### 2.3. Chemical Properties of the Bioplastics

**Figure 8:**
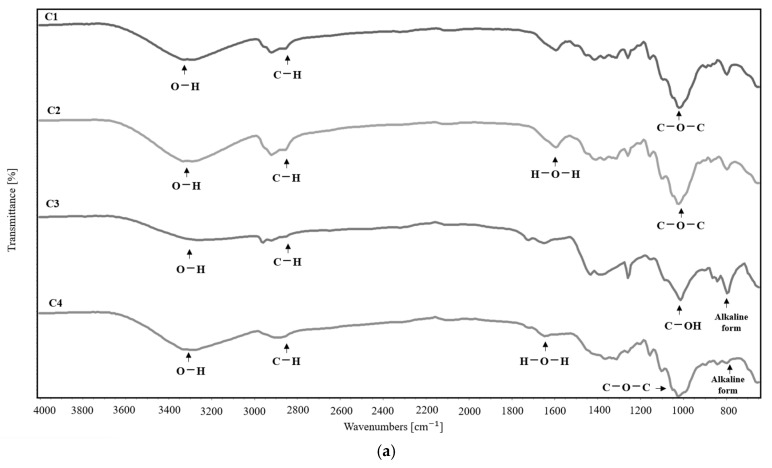

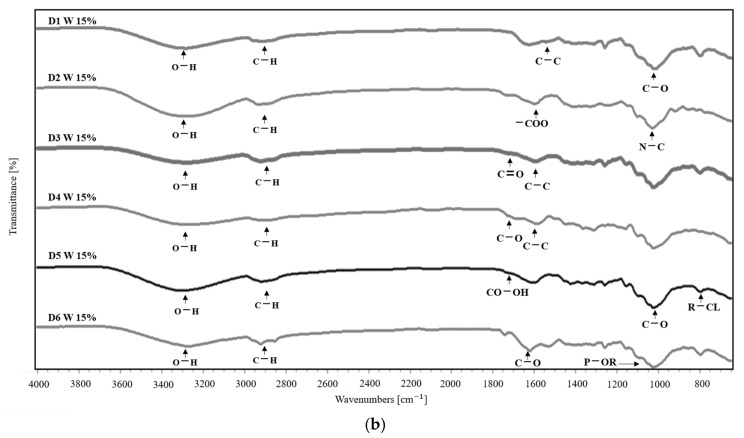
(**a**) FTIR spectra of bioplastic films used as controls: C1 (cellulose), C2 (cellulose + glycerol), C3 (cellulose + NaOH), and C4 (cellulose + NaOH + glycerol). (**b**) FTIR spectra of bioplastic films using natural partial dissolvent of cellulose D1 W, D2 W, D3 W, D4 W, D5 W, and D6 W at 15 wt.%.

The FTIR spectra of the cellulose bioplastics at 15 wt.% of natural dissolvent can be seen in Figure 8a. In the spectrum of C1 (cellulose), the O-H peaks (3300–3500 cm^−1^) usually show a shift toward lower frequencies compared with pure water due to the formation of hydrogen bridges between water and the hydroxyl groups of cellulose [35]. Also, in the range 1400–1200 cm^−1^, there is a small peak characteristic of the stretching vibration of C-H in cellulose. The sample C2 (cellulose + glycerol) registered C-O stretching in the 1125–1000 cm^−1^ region related to the alcohol of glycerol after a plasticizing reaction with cellulose [36]. The control C3 (cellulose + NaOH) registered a peak in the range of 700–650 cm^−1^ belonging to the COH functional group after adding NaOH [37]. The positive control C4 (cellulose + NaOH + glycerol) integrated the peaks of –OH, C-H, and CO; these results show the correct incorporation of the compounds in the bioplastic film.

The presence of the functional groups C=O, C-C, C-O, and COO- is shown, which correspond to the chemical structure of the solvent molecule D4 W. Therefore, the incorporation of the solvent extract into the structure of the films D4 W 15% is demonstrated. Then, the bioplastic formulated with the D5 W extract shows the peaks C-C, C-O, C-Cl, and C-Br, indicating the presence of the extract solution in the film. Finally, the presence of the peaks corresponding to the functional groups of P-H, N-H, and C-O is observed, as well as the functional groups of P-OR and CH_2_, characteristic of the D6 W extract. These functional groups demonstrate the molecule’s presence as a solvent in the bioplastics (Figure 8b).

The FTIR analysis of the bioplastic films with the natural extract D1 W and glycerol shows a significant difference in the functional groups when using bioplastic films with different concentrations from 0 to 15 wt.% compared with the negative control C1 (cellulose) and the positive control C4 (cellulose + NaOH + glycerol).

### 2.4. Surface Analysis of Bioplastics

A scanning electron microscope (SEM) was employed to acquire detailed information regarding the structure, morphology, size, shape, and surface modifications of the bioplastics [28,29]. The high magnification and resolution capabilities of the SEM facilitated a thorough examination and analysis of the bioplastic film surface. The results reveal distinct structural variations in both the cross-section and upper surface of the bioplastic, as illustrated in Figure S1 (see the Supplementary Materials). The SEM analysis of the film with the highest concentration of D1 W showed semi-organized fiber arrangements on the surface. The cross-sectional view revealed a semi-organized arrangement of fibers within the inner portion of the film. In contrast, the surface of the D2 W film appeared rough, and the cross-sectional analysis showed a disorganized arrangement of internal fibers.

The films prepared with D3 W exhibited a rough surface with noticeable pores in certain areas, as well as accumulations associated with the extract. The cross-sectional analysis revealed a disorganized distribution of internal fibers. In the case of D4 W, the SEM images showed a surface with hierarchical fibers and a limited presence of pores. However, the cross-sectional view indicated a disorganized distribution of fibers within the film, with no clear orientation. For D5 W, the results suggest some fibrillar intertwining and residual solvent on the surface, which may be related to the solubility of the extract. The cross-sectional analysis revealed a distinct internal distribution of components. Additionally, the images of the film with the D6 W extract showed a non-uniform fiber distribution and a porous structure. The surface appeared rough, and the fibers were oriented in a disorganized manner.

### 2.5. Biodegradability of Bioplastics

#### Degradation in Soil and Water

The biodegradability of the cellulose bioplastics, formulated with a natural solvent and glycerol, was assessed using the soil burial method [38]. Soil, being rich in microorganisms, facilitates plastic biodegradation more effectively compared with other environments such as water and air [38]. The bioplastics demonstrated a need for elevated temperatures and extended degradation periods to degrade efficiently. The rate of polymer biodegradation is influenced by the chemical nature of the polymer, environmental conditions, and soil composition.

**Figure 9:**
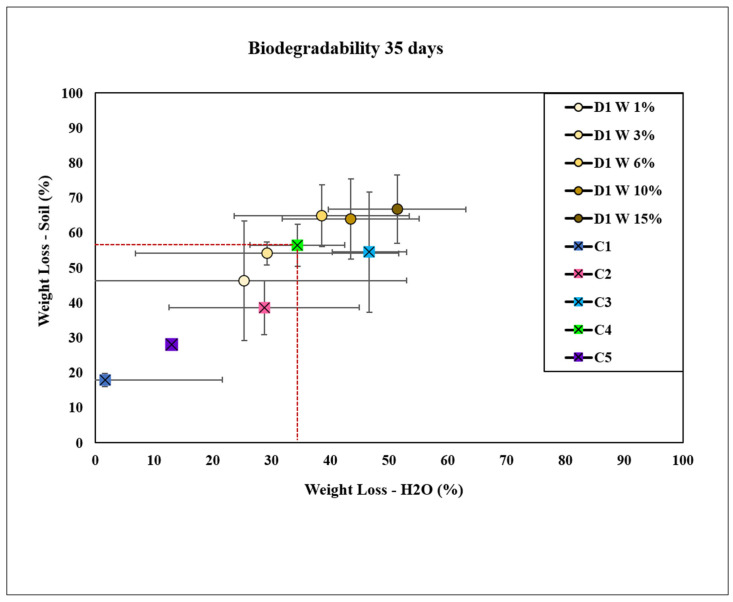
Bioplastics with plant extract D1 W degraded in soil and water at concentrations of 1, 3, 6, 10, and 15 wt.% compared with controls C1 (cellulose), C2 (cellulose + glycerol), C3 (cellulose + NaOH), C4 (cellulose + NaOH + glycerol), and C5 (bioplastic of avocado).

The results presented in Figure 9 demonstrate that cellulose bioplastics formulated with the plant extract D1 W at concentrations of 6, 10, and 15 wt.% exhibited accelerated degradation rates compared with the commercial control, C4. This observation suggests that incorporating the plant extract as a solvent at intermediate-to-higher concentrations enhances the biodegradability of cellulose bioplastics in both soil and water environments. By day 35, the bioplastics containing the plant extract showed significant disintegration. In contrast, the negative control, C1, exhibited the least weight loss in both soil and water. Bioplastics formulated without a conventional solvent, using only a traditional plasticizer (C2), demonstrated a slower degradation rate compared with films that included a traditional solvent (C3). Additionally, the avocado bioplastic (C5) was also evaluated.

The observed differences were statistically significant in both water and soil, indicating that the presence of the cellulose solvent, rather than the conventional plasticizer, is crucial for accelerating the degradation process. The extremely low *p*-values further confirm statistically significant differences between the groups in both water and soil degradation studies, underscoring that the various treatments or conditions substantially influenced weight loss due to biodegradation. Furthermore, the significant differences observed among the different concentrations of D1, as well as between the positive and negative controls, suggest that the concentration of D1 substantially impacted the biodegradation rates.

**Figure 10:**
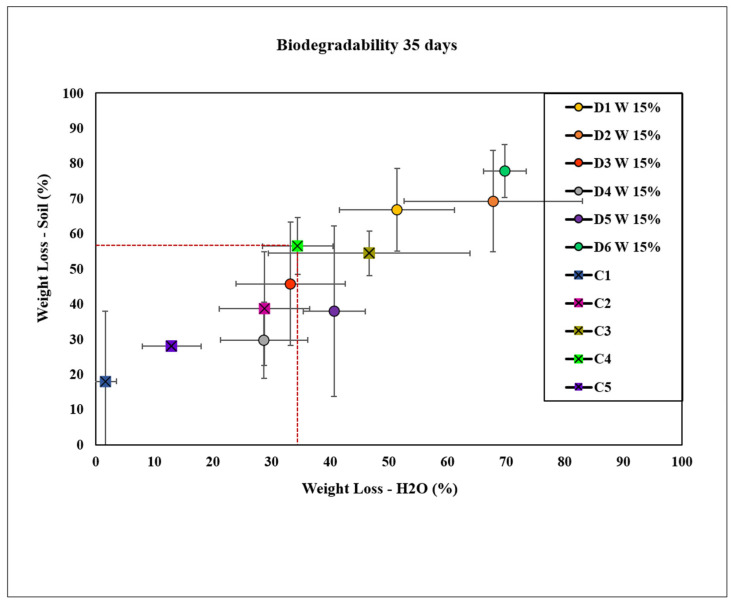
Degradation in soil and water of bioplastics with plant extracts D1 W, D2 W, D3 W, D4 W, D5 W, and D6 W at a concentration of 15 wt.% compared with controls C1 (cellulose), C2 (cellulose + glycerol), C3 (cellulose + NaOH), C4 (cellulose + NaOH + glycerol), and C5 (bioplastic of avocado).

The results presented in Figure 10 illustrate the degradation values of bioplastics containing 15 wt.% of extracts D1 W, D2 W, D3 W, D4 W, D5 W, and D6 W after 35 days. Bioplastic films incorporating extracts D3 W and D4 W exhibited relatively lower weight loss in both soil and water environments compared with the commercial control, C4. This suggests that these specific extract formulations may enhance the durability of bioplastics under these conditions. Conversely, bioplastic films containing extract D5 W displayed a distinct degradation pattern, with slower degradation in soil relative to C4 but accelerated degradation in water. This contrasting behavior indicates that the effect of extract D5 on bioplastic degradation was dependent on the environmental medium. Additionally, bioplastic films containing extracts D1 W, D2 W, and D6 W demonstrated more rapid degradation rates in both soil and water environments. Although we did not have evidence of abrine in the extract used for bioplastic D2W, it is possible that the extract had other molecules that could act as a dissolvent for cellulose and accelerate the degradation of D2W.

Tukey’s HSD test for weight loss in water revealed a significant difference between C1 and D1. Additionally, C1 had a significantly different mean compared with all other groups (D1 to D6, C2, C3, C4, and C5). Extract D2 exhibited significant differences from most other groups, with the exception of D6. Extracts D3, D4, and D5 displayed varied significance when compared with different groups. This variability underscores the complex nature of factors influencing the degradation kinetics of bioplastics.

In terms of weight loss in soil, C1 showed significant differences from all other groups except D4 and C5, indicating that the negative control experienced much lower weight loss compared with most other treatments and controls. The partial dissolution of cellulose occurred during the mixing step with the base (NaoH in the plant extract). It did not occur in C1 and C2, which were our controls without the base, and this can explain the slower degradation of the samples without the base. Extract D1 was significantly different from D3, D4, D5, and C1. Extract D2 also showed substantial differences from D3, D4, D5, and C1, but not from D6. Extract D3 exhibited significant differences from D4, D5, and D6. Extracts D4 and D5 showed substantial differences compared with multiple groups, reflecting their varied effectiveness compared with other treatments and controls. Extract D6 demonstrated significant differences from C1, C2, C3, C4, and C5, suggesting it had the highest weight loss among the treatments.

### 2.6. Water Uptake of Bioplastics

**Figure 11:**
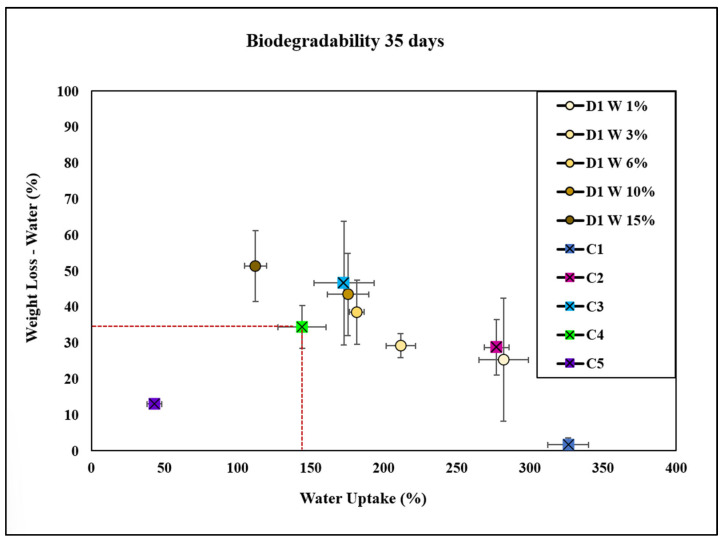
Water uptake and degradation of bioplastics with the plant extract D1 W at concentrations of 1, 3, 6, 10, and 15 wt.% compared with controls C1 (cellulose), C2 (cellulose + glycerol), C3 (cellulose + NaOH), C4 (cellulose + NaOH + glycerol), and C5 (bioplastic of avocado).

**Figure 12:**
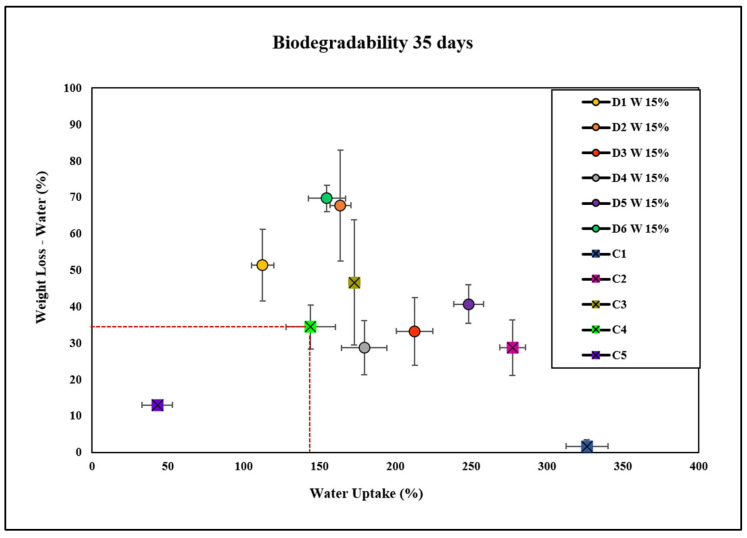
Water uptake and degradation of bioplastics with the plant extracts D1 W, D2 W, D3 W, D4 W, D5 W, and D6 W at a concentration of 15 wt.% compared with controls C1 (cellulose), C2 (cellulose + glycerol), C3 (cellulose + NaOH), C4 (cellulose + NaOH + glycerol), and C5 (bioplastic of avocado).

Water absorption is defined as the amount of water taken up by a bioplastic. It is calculated as the ratio of the weight of water absorbed to the weight of the dry material [11]. The results presented in Figure 11 and Figure 12 indicate that an increased amount of natural fiber leads to a higher degradation rate. This occurs because the natural fiber absorbs water, which accelerates the hydrolytic degradation process [39]. Assessing water absorption in cellulose bioplastic films is crucial for evaluating the stability of these films in aqueous environments, which impacts their long-term performance.

The C4 bioplastic film exhibited a water uptake of 150% and a weight loss of 35%. In contrast, the C1 bioplastic film showed a higher water uptake of 325% (see Figure 11). This difference suggests that the inclusion of a conventional plasticizer and cellulose solvent reduced the water uptake. This effect can be attributed to the interaction between glycerol and NaOH, which likely altered the structural properties of the bioplastic film. Specifically, the plasticizer increased the flexibility of the film, while the cellulose solvent facilitated the partial dissolution and dispersion of cellulose molecules, resulting in a more compact and less porous structure [39]. Consequently, the film exhibited reduced water uptake compared with the control cellulose C1 due to the decreased availability of free spaces within its structure for water molecules to penetrate and be absorbed, demonstrating that the alkaline solution and the natural extract were required to form the bioplastic.

Figure 9 illustrates that at lower concentrations of D1 W, the water uptake and degradation rates were lower than those observed for C4. In contrast, at the highest concentration of 15% extract D1 W, the water uptake was lower than that of C4, and the degradation in water was more efficient than that of C4. This result can be attributed to the increased concentration of active components in the extract, which likely enhanced the hydrophobicity of bioplastic film. The higher concentration of extract D1 W may also have introduced more effective degradation-promoting components, leading to improved degradation rates in water compared with C4. Additionally, C1 exhibited significantly higher water uptake than all other groups. D1 W 15% demonstrated a significantly lower water uptake than all other groups except C5. Meanwhile, C1 had significantly lower weight loss compared with all other groups. D1 W 15% exhibited significantly higher weight loss than C1 but lower than certain other concentrations, such as D1 W 6% and D1 W 10%. D1 W 6% and D1 W 10% showed significantly higher weight loss compared with C1, C2, and C5. Comparisons between D1 W 1%, D1 W 3%, and the other concentrations showed variability in weight loss, but the differences were not consistently significant.

The negative control C1 group exhibited significantly higher water uptake compared with all other groups. The D1 W 15% group demonstrated significantly lower water uptake compared with C1, C2, and D3, but significantly higher water uptake compared with D4, D5, D6, and C4. D2 W 15% showed a significantly higher water uptake than D1 W 15%, D4, D5, D6, and C4, but lower than C1. The D3 W 15% group had the highest water uptake among the D1 concentrations, significantly higher than all other groups except C1. The D4 W 15% group exhibited significantly higher water uptake compared with D1 W 15%, D5, D6, and C4, but lower than D2 W 15% and D3 W 15%. D5 W 15% showed a significantly higher water uptake than D1 W 15%, D2 W 15%, D4 W 15%, D6 W 15%, and C4, but not compared with C1. D6 W 15% had a significantly higher water uptake compared with D1 W 15%, D2 W 15%, D4 W 15%, and C4, but lower than C1. Overall, C1 exhibited the highest water uptake among all groups, with significant differences from every other group. C2 and C3 also had significantly higher water uptake compared with the D1 concentrations and C4. The principal findings about the biodegradability of the bioplastics studied are presented in Table 4.

The D1 W 15% group showed significant differences from other concentrations (D3 W 15%, D4 W 15%, and D5 W 15%), although these differences were less pronounced. D2 W 15% and D6 W 15% showed the highest weight loss among D1 concentrations and compared with several controls. D1 W 15% showed lower weight loss compared with higher D1 concentrations (D2 W 15% and D6 W 15%) but higher compared with C1. C1 consistently showed the lowest weight loss, significantly different from all other groups, highlighting its role as the negative control. C5 had the lowest weight loss among all controls and D1 concentrations, indicating it might be an outlier or less effective in weight loss. C2 and C3 exhibited higher weight loss compared with C1 and C4, but this was not as high as that of D2 W 15% and D6 W 15%.

Bioplastic films containing extracts D1 W, D6 W, and D2 W exhibited similar levels of water uptake and higher degradation rates compared with the positive control, C4. This can be attributed to the composition of these extracts, which likely contained molecules that increased the hydrophilicity of the films, allowing for water molecules to penetrate more easily and accelerate degradation. Conversely, films with extracts D3 W and D5 W showed similar weight loss but higher water uptake compared with C4, indicating that these extracts enhanced the water absorption capacity of the bioplastic, leading to swelling without necessarily increasing degradation. The film with extract D4 W had lower degradation rates but higher water uptake than C4, suggesting that D4 W may contain components that inhibit degradation while promoting water absorption, potentially acting as barriers to degradation or altering the film’s structural integrity.

### 2.7. pH Change in Cause Medium

The effects of the water pH and immersion time on the water absorption behavior and mechanical properties of the bioplastic films were studied using a benchtop pH meter (Benchtop pH Meter APERA Instrument). The six molecules selected by computational screening had acid dissociation constant (pKa) values that were generally basic, so the changes in the water pH over time were monitored during degradation tests. The results are presented in Figure S2 (Supplementary Materials). Samples C3 and C4 showed significantly different pH variations compared with the other samples, exhibiting a more alkaline pH during the water degradation test. This difference was due to the inclusion of sodium hydroxide in these formulations, which acted as both a conventional cellulose solvent and a standard alkalizing agent in this study [7].

In contrast, bioplastics C1 and C2 did not show significant pH fluctuations. Among the samples containing plant extracts, no significant pH differences were observed compared with the positive control, except for D1 W at a concentration of 15 wt.%. However, the pH change for these extracts was less than that observed for C3 and C4. Plant extracts often contain organic acids and other compounds that act as buffering agents, stabilizing the pH. Thus, the plant extract solvent in D1 W at 15 wt.% likely had a less pronounced alkalizing effect on the solution, resulting in a lower pH change compared with the NaOH-containing formulations.

The statistical test revealed significant differences in pH levels across various time points, indicating that the pH fluctuated depending on the extract concentration and immersion duration. The control samples C3 and C4, containing NaOH, showed higher pH variability, maintaining an elevated pH due to their strong alkalizing effect. The lower extract concentrations (1% to 6%) exhibited moderate pH changes, suggesting a minimal impact due to their lower chemical activity or buffering capacity. In contrast, higher extract concentrations (10% and 15%) displayed marked pH increases over time, likely due to a cumulative interaction with water. Each extract demonstrated a distinct pH profile, reflecting its unique chemical composition. Some extracts, such as D2 W and D5 W, showed stable pH levels, possibly due to buffering agents. In contrast, control samples C1 and C2, containing commercial bioplastic and glycerol, showed minimal pH alterations, consistent with expectations for non-alkalizing components.

## 3. Materials and Methods

### 3.1. Computational Screening

Computational methods have revolutionized problem solving across diverse fields by leveraging advanced high-performance computing systems. The ability to process vast quantities of data and construct models predicting molecular properties or biological activities based on structure–property relationships is notable for several reasons: Firstly, these studies offer a more cost-effective and time-efficient approach than traditional experimental methods, accelerating the discovery and development of new molecules with specific desired properties. The popularity of quantitative structure–activity relationship (QSAR) and quantitative structure–property relationship (QSPR) methods stems from their established reliability and effectiveness in predicting molecular behavior [40].

The decision to use compounds from the Analyticon MEGx database, available at https://ac-discovery.com (accessed on 15 February 2024), for screening was likely based on the database containing a diverse and representative set of compounds relevant to this research objective. The choice to use two specific datasets, MEGx_1 and MEGx_2, from the database was based on the characteristics of the compounds included. MEGx_1, with its larger size of 6348 compounds, provides a comprehensive dataset for a broad screening of properties or activities. Meanwhile, MEGx_2, consisting of 414 compounds, is more focused and curated, possibly containing compounds of specific interest or relevance to this research.

The aim of using the software program PaDEL, version 2.21, a descriptor for substructure fragment analysis with a specific fingerprint, SubFPC12, associated with the OH group, was to identify molecular fragments or patterns in the examined compounds similar to conventional solvents [33]. This analysis provided insights into the structural features of the compounds relevant to their properties, such as solubility or toxicity. Furthermore, the evaluation of acute oral toxicity using the software program (developed in Java 1.8) indicated a focus on assessing the potential toxicity of the compounds of this research [41]. The EPA (Environmental Protection Authority) and GHS (Globally Harmonized System) classification systems provided a comprehensive approach to toxicity evaluation, considering both environmental and human health hazards.

### 3.2. Extract Source Screening

The selection of six natural sources was based on computational screening and the literature reporting the presence of the identified molecules. Table 5 presents the compound name, chemical classification, molecular weight, pKa, and natural source of the molecules. After source identification, quinoa (*Chenopodium quinoa*), guayaba (*Psidium guajava*), palo santo (*Bursera graveolens*), Ivory Coast almond (*Terminalia ivorensis*), cacalosuchil (*Plumeria rubra*), and soya (*Glycine max*) were obtained from the local supermarket.

### 3.3. Other Reagents

The hemp (*Cannabis sativa* L.) biomass used in this study was acquired from CannAndes S.A. (Tabacundo, Ecuador). Merck-Milipore (Darmstadt, Germany) supplied sodium hydroxide, ethanol, methanol, and glycerol.

### 3.4. Preparation of Cellulose Bioplastic

#### 3.4.1. Cellulose Extraction

Cellulose was extracted from hemp through a series of treatments, including alkalization, acid hydrolysis, and bleaching [42]. Initially, the hemp biomass was subjected to crushing and cleaning to eliminate any contaminants. The resultant residue was then treated with hydrochloric acid. Following this, the biomass underwent alkalization using sodium hydroxide. Subsequently, the biomass was bleached with chlorine for 24 h at an ambient temperature. The final residue was then thoroughly washed, dried at room temperature, and then lyophilized. This method effectively yielded a high purity and quantity of cellulose by removing non-cellulosic components. The cellulose was then stored at room temperature. The yield of hemp cellulose was calculated as follows [43]:

(1) $$Cellulose\;yield\left(\%\right)=\frac{Mass\;of\;cellulose\;obtained}{Initial\;mass\;of\;hemp}\times 100$$

#### 3.4.2. Obtention of Plant Extract

The sample collection was conducted based on the results of computational screening. Biomass was obtained from six distinct sources for the extraction of each molecule. These samples were initially stored at room temperature (25 °C), then crushed into a flour-like powder, packaged, and stored. Two of the biomass samples were washed, dried in an oven at 40 °C for 48 h and then crushed and subsequently stored at room temperature. Following collection, the maceration method (see Table 6) was employed for extraction due to the coarse powder consistency of the biomass material [44].

Distilled water (W) was employed as the solvent for molecule extraction. It was added to the biomass samples, with the maceration period varying from 24 h to 5 days, depending on the source, and continuous agitation was maintained [45]. Following the maceration period, the samples were subjected to centrifugation at 6000 rpm for 30 min. The supernatant was collected, filtered, frozen at −80 °C, and subsequently lyophilized. The resulting powdered extracts were stored and labeled as D1 W, D2 W, D3 W, D4 W, D5 W, and D6 W.

#### 3.4.3. Cellulose Bioplastics

The general formulation for the cellulose bioplastics comprised glycerol as a standard plasticizer, plant extract, hemp cellulose, and water (see Table 7). For comparison purposes, the control sample C1 served as the negative control, while C4 acted as the positive control. Each sample was magnetically stirred for 60 min at 80 °C. Following this, the mixture was subjected to hot pressing (Dabpress, Shenzhen, China) at 240 °C for 10 min to form the bioplastic films, which were then cut into circular discs with a diameter of 1.791 cm [46]. The films were subsequently dried at room temperature to complete the bioplastic formation. The glycerol content was maintained consistently across all samples.

### 3.5. Characterizations of Plant Extract

#### 3.5.1. Fourier-Transform Infrared Spectroscopy of Plant Extract

The Fourier-transform infrared spectroscopy (FTIR) spectrum of each extract was recorded in the infrared region, covering wavelengths from 4000 and 650 cm^−1^ [47]. This method was employed to identify functional groups. A small amount of plant extract was analyzed using the Cary 630 Fourier-transform infrared spectrometer (Agilent Technologies, Stockport, UK) to elucidate the structural characteristics and the intensity of the absorption spectra related to molecular composition or chemical functional groups [48]. The resulting spectral data were then documented.

#### 3.5.2. Phytochemical Analysis

A preliminary qualitative phytochemical analysis was performed to identify secondary metabolites in aqueous extracts from six distinct sources. This study specifically focused on detecting amino acids, proteins, and terpenoids. For this purpose, 100 mg of the powdered plant samples was dissolved in 5 mL of type 1 water (analytical grade). Each extract was then evaluated for the presence of reducing sugars, proteins, amino acids, terpenoids, diterpenes, saponins, glycosides, alkaloids, coumarins, lactones, flavonoids, phenols, and quinones [49].

#### 3.5.3. Thin-Layer Chromatography

The powdered extract was employed for thin-layer chromatography (TLC) profiling. Both the samples and standards were diluted in type 1 water. The prepared aqueous extract was then shaken and filtered to obtain a particle-free solution, which was used for TLC analysis on 20 × 20 cm aluminum sheets coated with silica gel 60 F_254_ [50]. The sample was applied to the plate by a capillary. The plates, loaded with samples, were developed in a chamber containing a solvent mixture with a specific solvent system (see Table 8). TLC was performed on extracts and pure samples of the target compound. After development, the plates were dried and visualized in a UV chamber at 254 nm. Iodine and other revelators were used, and photographs of the plates were taken. The Rf values of each spot were calculated using Equation (2):

(2) $$R_{f}\;value=\frac{distance\;molecule\;traveled}{distance\;solvent\;traveled}$$

### 3.6. Characterizations of Bioplastics

#### 3.6.1. Fourier-Transform Infrared Spectroscopy of Bioplastics

The FTIR spectra were used to identify the functional groups of the cellulose bioplastics in the FTIR Cary 630 Fourier-transform infrared spectrometer (Agilent Technologies, Stockport, UK) [51]. The films were placed in a sample holder and scanned in the 4000–600 cm^−1^ range.

#### 3.6.2. Scanning Electron Microscope (SEM)

The surface morphology of the bioplastic film was checked by using a scanning electron microscope (JEOL USA Inc, Peabody, MA, USA). The structure of the cross-section and upper surface was analyzed [52]. The sample was examined under magnifications such as 50×, 100×, and 400×.

#### 3.6.3. Degradation in Water Test

The solubility of the cellulose bioplastics was analyzed by immersing the samples in water [42]. The samples, which were prepared as circular discs with a diameter of 1.791 cm, were initially dried in an oven at 40 °C for two hours. Each sample was weighed to determine its initial mass (M_1_) and then placed in a plastic cup containing 100 mL of distilled water. The samples were left at room temperature for 6 h, 1 day, 3 days, 8 days, 21 days, and 35 days. After these time intervals, the samples were dried in an oven (RebelK, RebelK PCD-E9000, Washington, DC, USA) at 40 °C for 24 h. The final samples were weighed for their final weight (M_2_). The percentage of solubility in water was calculated using Equation (3):

(3) $$H_{2}O\;Degradation=\frac{\left(M_{1}-M_{2}\right)}{M_{1}}*100$$

#### 3.6.4. Absorption of Water Test

The water absorption by the bioplastics was determined using a slightly modified ASTM D570-98 method [53]. The bioplastic samples, prepared as circular discs with a diameter of 1.791 cm, were initially dried in an oven at 40 °C for 2 h to obtain their dry weight (M_1_). The samples were then immersed in 100 mL of distilled water at room temperature for 6 h, 1 day, 3 days, 8 days, 11 days, and 35 days. The bioplastics were retrieved by filtering the water and their final weight (M_2_). The absorption of water was found using the following Equation (4):

(4) $$H_{2}O\;Absorption=\frac{\left(M_{2}-M_{1}\right)}{M_{1}}*100$$

#### 3.6.5. Biodegradability Test

The biodegradation of the cellulose bioplastics was assessed using the soil burial method [53]. The samples, prepared as circular discs with a diameter of 1.8 cm, were initially weighed (M_1_) using a digital balance. The samples were buried in garden soil and placed in plastic cups. They were maintained in these cups for 3, 8, 21, and 35 days, with the soil moisture and temperature controlled to mimic natural conditions: the temperatures ranged from ambient conditions at night to 37 °C during the day. After the designated periods, the samples were retrieved from the soil, cleaned of residual soil, and dried in an oven at 37 °C for 24 h. The final weight (M_2_) was then recorded, and the percentage of biodegradation was calculated using Equation (5):

(5) $$Degradation\;in\;Soil=\frac{\left(M_{1}-M_{2}\right)}{M_{1}}*100$$

#### 3.6.6. Column Chromatography Fractionation

The D1 W plant extract was subjected to silica gel column chromatography to isolate phytoconstituents of interest [47]. The elution systems employed included butanol, acetic acid, and water. A concentrated aqueous extract of 30 mg was fractionated using column chromatography on silica gel (60–120 mesh) [54]. The fractions were collected and subjected to further analysis.

#### 3.6.7. Metabolomic Profile

Data acquisition was performed by Waters model Acquity I-Class Ultra Performance Liquid Chromatography (Waters, Milford, MA, USA) coupled to Tandem Mass Spectrometry (UPLC-MS/MS) equipment for LC modules coupled to a XEVO G2-XS QTOF quadrupole time-of-flight mass spectrometer. Chromatographic separation was achieved using a Waters ACQUITY UPLC CSH C18 column (50 × 3 mm), with a 1.7 μm particle size. The mobile phase was 0.1% formic acid in water (Solvent A) and acetonitrile (Solvent B). The flow rate and sample injection volume were set at 0.5 mL/min and 5 μL, respectively. The solvent gradient program was set as follows: 0.0–0.5 min, 1–99% B; 0.5–8.0 min, 99–100% B; 8.0–9.0 min, 100% B; 9.00–9.01 min, 100–1% B; and 9.01–10 min, 1% B. Electrospray ionization (ESI) was performed in positive mode for MS data acquisitions. Unified tune parameters were set for the MS and fast data-dependent acquisition (Fast DDA) experiments: the capillary voltage was kept at 0.5 kV; the source temperature was 120 °C; the desolvation temperature was 450 °C; the nitrogen desolvation gas flow rate was 900 L h^−1^; the cone gas flow rate was kept at 0 L h^−1^, and argon was used as the collision gas. Both MS and Fast DDA data were acquired in resolution mode. The data were calibrated using an external reference (Waters Corporation, Milford, MA, USA, LockSpray^TM^) by the constant infusion of a leucine–enkephalin solution (1 ng/L) at a 10 μL/min flow rate. Data acquisition was performed using the MassLynx V4.1 software (Waters, Milford, MA, USA). In Fast-DDA mode, the mass range of the full MS and MS2 scans were *m*/*z* 100–1200 and 50–1200 Da, respectively.

#### 3.6.8. Statistical Analysis

After the samples were characterized, the results of the physiochemical properties were analyzed, tabulated, and represented graphically. These results were analyzed statistically using Microsoft Excel 2019.

## 4. Conclusions

In this study, we investigated the feasibility of utilizing plant extract-based solvents for the partial dissolution of cellulose, a crucial step in the production of bioplastics. Our findings indicate that these plant extract-based solvents, derived from renewable plant sources, demonstrated notable effectiveness in partially dissolving hemp cellulose for the preparation of bioplastic, suggesting their potential utility in sustainable bioplastic production. This research successfully demonstrated the synthesis of bioplastics using a dissolvent obtained from plant extract-based solvents. The resulting bioplastics exhibited promising chemical and physical properties, making them viable candidates for a range of applications. This suggests a significant step forward in the development of environmentally friendly alternatives to traditional petroleum-based plastics for single-use plastic products. Future studies should aim to optimize these aspects to make plant extract-based solvents even more practical and competitive in large-scale bioplastic production.

## Figures and Tables

**Table 1:** Phytochemical profiles of the molecules in the extracts D1 W, D2 W, D3 W, D4 W, D5 W, and D6 W.

Phytochemical Analysis	Test	D1 W	D2 W	D3 W	D4 W	D5 W	D6 W	Ref.
Reducing sugars	Benedict’s test	X	X	X	X	X	-	[18]
	Fehling’s test	X	X	X	-	X	X	[18]
Proteins and amino acids	Ninhydrin test	X	-	X	-	-	X	[19]
	Xanthoproteic test	X	X	X	X	X	X	[19]
Proteins	Biuret test	-	-	X	-	-	X	[20]
Terpenoids	Salkowski test	X	X	X	X	X	-	[19]
Diterpenes	Copper acetate test	X	X	X	-	X	-	[21]
Saponins	Foam test	-	X	X	X	X	X	[19]
Glycosides	10% NaOH test	X	X	-	-	X	-	[21]
	Keller–Kilani test	-	X	X	-	X	-	[21]
Alkaloids	Dragendroff test	-	-	X	X	X	-	[22]
Coumarins and lactones	NaOH test	X	-	-	-	-	-	[23]
Flavonoids	Alkaline reagent test	X	X	X	X	-	-	[21]
Phenols	Ferric chloride test	X	X	-	X	X	-	[24]
Quinones	Bontrager test	-	X	X	X	X	-	[25]
	Sulfuric acid test	-	X	X	-	-	X	[25]

(-) absence; (X) presence.

**Table 2:** Mobile phase, Rf values, and solvent developers set for each crude extract in TLC analysis.

Crude Extract	Mobile Phase	Retention Factor (Rf)	Developer
D1 W	n-butanol/acetic acid/water	0.65732	Ninhydrin 2% + ethanol
D2 W	n-butanol/acetic acid/water	0.59523	Ninhydrin 2% + ethanol
D3 W	Toluene/ethyl acetate	0.40000	Vanillin sulfuric acid ethanol
D4 W	Hexane/acetone	0.53703	Iodine
D5 W	Chloroform/ethanol	0.38596	Iodine
D6 W	Toluene/ethyl acetate	0.78947	Vanillin sulfuric acid ethanol

**Table 3:** Identification and characterization of metabolites via mass spectrometry: analysis of phenylalanine (D1 W) and 20-hydroxyecdysone.

IL *	RT (min.)	[M + H]^+^ (*m*/*z*)	Molecular Formula	Error (ppm)	MS2 Score *	Identified Metabolite Name
1	1.61	(120.0803 fragment ion)	C_9_H_11_NO_2_	−3.33	-	Phenylalanine
2	2.92	445.294 [M-2H2O + H]+	C_27_H_44_NO_7_	1.79	0.72	20-hydroxyecdysone

* IL—identification level according to [33].

**Table 4:** Biodegradability of bioplastics, including principal conclusions about degradation rate, water uptake, and certain observations.

Bioplastic Type	Composition	Degradation Rate (%)	Water Uptake (%)	Observations
C1	Cellulose (negative control)	Lowest	Highest (325%)	Exhibits the least weight loss in both soil and water, indicating lower biodegradability. Shows the highest water uptake among all groups.
C2	Cellulose + glycerol	Moderate	Moderate	Demonstrates a slower degradation rate compared with C3. Shows higher water uptake than D1 and D6 at 15 wt.%.
C3	Cellulose + NaOH	Moderate	Moderate	Slightly faster degradation than C2. Exhibits higher water uptake compared with C4 but lower than C1.
C4	Cellulose + NaOH + glycerol (commercial control)	Faster	Lower (150%)	Exhibits a faster degradation rate compared with C1, C2, and C3. Lower water uptake compared with C1 but higher than C5.
C5	Bioplastic of avocado	Lower	Lowest	Shows the lowest weight loss among all controls and D1 concentrations, indicating low biodegradability. Water uptake is lower compared with other groups.
D1 W 1%	Plant extract D1 W (1 wt.%)	Lower	Higher	At low concentrations, it exhibits reduced biodegradability and higher water uptake compared with D1 W 15%.
D1 W 3%	Plant extract D1 W (3 wt.%)	Lower	Higher	Similar to D1 W 1%, with moderate water uptake and degradation rates. Differences in weight loss compared with D1 W 6% and 10% were not consistently significant.
D1 W 6%	Plant extract D1 W (6 wt.%)	Higher	Moderate	Higher degradation rate compared with D1 W 1% and 3%. Water uptake is moderate, significantly higher than C1.
D1 W 10%	Plant extract D1 W (10 wt.%)	Higher	Moderate	Has a degradation rate similar to D1 W 6%, with higher weight loss compared with C1, C2, and C5. Moderate water uptake.
D1 W 15%	Plant extract D1 W (15 wt.%)	Highest	Lower	Exhibits the highest degradation rates in both soil and water, especially compared with C1. Lower water uptake than C1 and D1 W 1%.
D2 W 15%	Plant extract D2 W (15 wt.%)	High	Moderate	High degradation rates in both soil and water. Water uptake is higher than D1 W 15%, D4, D5, D6, and C4, but lower than C1.
D3 W 15%	Plant extract D3 W (15 wt.%)	Moderate	High	Exhibits relatively lower weight loss but higher water uptake compared with C4. High water uptake indicates enhanced water absorption capacity without necessarily increasing degradation.
D4 W 15%	Plant extract D4 W (15 wt.%)	Lower	High	Lower degradation rates but higher water uptake than C4, suggesting components in D4 W may inhibit degradation while promoting water absorption.
D5 W 15%	Plant extract D5 W (15 wt.%)	Variable	High	Show slower degradation in soil but accelerated in water compared with C4. It indicates that D5 W effect is environment dependent. High water uptake but does not necessarily increase degradation.
D6 W 15%	Plant extract D6 W (15 wt.%)	High	Moderate	High degradation rates similar to D1 W 15% and D2 W 15%. Water uptake is higher than C4 but lower than D3 W 15%.

**Table 5:** Molecules identified by computational screening.

Code	Compound Name	Chemical Class	Molecular Weight (g/mol)	OH Groups	Predicted pKa	Source
D1	Phenylalanine	Amino acids and peptides	165.192	0	9.48	Quinoa
D2	Abrine	Amino acids and peptides	218.256	0	9.34	Guayaba
D3	Alpha-Boswellic acid acetate	Terpenoids	498.748	0	9.06	Palo santo
D4	Bellericagenin B	Terpenoids	520.707	5	9.02	Ivory Coast almond
D5	Obtusol	Terpenoids	442.728	2	9.01	Cacalosuchil
D6	1-linoleoyl-sn-glycerol-3-phosphorylcholine	Aliphatic natural product	426.729	1	9.11	Soya

**Table 6:** Maceration method quantities.

Source	Amount Biomass (g)	Water (mL)	Time
Quinoa	200	1000	1 day
Guayaba	280	350	1 day
Palo santo	125	500	7 days
Ivory Coast almond	50	500	7 days
Cacalosuchil	50	500	7 days
Soya	200	1000	1 day

**Table 7:** Cellulose bioplastic formulations with plant extract. C1: negative control; C2: conventional plasticizer control; C3: conventional dissolvent control; C4: positive control.

Code	Natural Extract	Hemp Cellulose (g)	NaOH (g)	Glycerol (g)	Water (mL)
D1 W, D2 W, D3 W, D4 W, D5 W, D6 W	1% (0.1 g), 3% (0.3 g), 6% (0.6 g), 10% (1.0 g), 15% (1.5 g)	1	-	1	10
C1	-	1	-	-	10
C2	-	1	-	1	10
C3	-	1	0.6	-	10
C4	-	1	0.6	1	10

**Table 8:** Thin-layer chromatography procedure for the crude extracts with the detailed mobile phase and their proportions.

Crude Extracts	Mobile Phase	Secondary Metabolite	Solvent System
D1 W	n-butanol/acetic acid/water	Amino acid	3:3:1
D2 W	n-butanol/acetic acid/water	Amino acid	3:3:1
D3 W	Toluene/ethyl acetate	Terpenoids	9.3:0.7
D4 W	Hexane/acetone	Terpenoids	2:1
D5 W	Chloroform/ethanol	Terpenoids	10:1
D6 W	Toluene/ethyl acetate	Aliphatic natural product	9.3:0.7

## Data Availability

The data are contained within this article.

## Supplementary Materials

The following supporting information can be downloaded at https://www.mdpi.com/article/10.3390/molecules30132752/s1. Figure S1. SEM images of the bioplastic films of quinoa (D1 W), guayaba (D2 W), palo santo (D3 W), Ivory Coast almond (D4 W), cacalosuchil (D5 W), and soya (D6 W) at the highest concentration of 15 wt.% at different magnifications; Figure S2. pH change in degradation in water test at 0 h, 6 h, 1 day, 3 days, 8 days, 21 days, and 35 days of the bioplastics films with plant extract and conventional plasticizers.

## References

[1] Kong U., Rawi N.F.M., Tay G.S. (2023). The Potential Applications of Reinforced Bioplastics in Various Industries: A Review. Polymers.

[2] Ramadan M., Cooper B., Posnack N.G. (2020). Bisphenols and phthalates: Plastic chemical exposures can contribute to adverse cardiovascular health outcomes. Birth Defects Res..

[3] Kumar R., Verma A., Shome A., Sinha R., Sinha S., Jha P.K., Kumar R., Kumar P., Shubham, Das S. (2021). Impacts of Plastic Pollution on Ecosystem Services, Sustainable Development Goals, and Need to Focus on Circular Economy and Policy Interventions. Sustainability.

[4] Rosenboom J.-G., Langer R., Traverso G. (2022). Bioplastics for a circular economy. Nat. Rev. Mater..

[5] Narancic T., Cerrone F., Beagan N., O’connor K.E. (2020). Recent Advances in Bioplastics: Application and Biodegradation. Polymers.

[6] Wang B.-T., Hu S., Yu X.-Y., Jin L., Zhu Y.-J., Jin F.-J. (2020). Studies of Cellulose and Starch Utilization and the Regulatory Mechanisms of Related Enzymes in Fungi. Polymers.

[7] Acharya S., Liyanage S., Parajuli P., Rumi S.S., Shamshina J.L., Abidi N. (2021). Utilization of Cellulose to Its Full Potential: A Review on Cellulose Dissolution, Regeneration, and Applications. Polymers.

[8] Zhou J., Zhang L. (2000). Solubility of Cellulose in NaOH/Urea Aqueous Solution. Polym. J..

[9] Wu C., Li J., Zhang Y., Li X., Wang S., Li D. (2023). Cellulose Dissolution, Modification, and the Derived Hydrogel: A Review. ChemSusChem.

[10] Gondhalekar S.C., Pawar P.J., Dhumal S.S., Thakre S.S. (2019). Mechanism of xanthation reaction in viscose process. Cellulose.

[11] Przypis M., Wawoczny A., Gillner D. (2023). Biomass and Cellulose Dissolution—The Important Issue in Renewable Materials Treatment. Appl. Sci..

[12] Hidayati S., Zulferiyenni, Maulidia U., Satyajaya W., Hadi S. (2021). Effect of glycerol concentration and carboxy methyl cellulose on biodegradable film characteristics of seaweed waste. Heliyon.

[13] Ben Z.Y., Samsudin H., Yhaya M.F. (2022). Glycerol: Its properties, polymer synthesis, and applications in starch based films. Eur. Polym. J..

[14] Wang H., Li H., Lee C.K., Nanyan N.S.M., Tay G.S. (2024). A systematic review on utilization of biodiesel-derived crude glycerol in sustainable polymers preparation. Int. J. Biol. Macromol..

[15] Benitez J.J., Florido-Moreno P., Porras-Vázquez J.M., Tedeschi G., Athanassiou A., Heredia-Guerrero J.A., Guzman-Puyol S. (2024). Transparent, plasticized cellulose-glycerol bioplastics for food packaging applications. Int. J. Biol. Macromol..

[16] Santana R.F., Bonomo R.C.F., Gandolfi O.R.R., Rodrigues L.B., Santos L.S., Pires A.C.d.S., de Oliveira C.P., Fontan R.d.C.I., Veloso C.M. (2018). Characterization of starch-based bioplastics from jackfruit seed plasticized with glycerol. J. Food Sci. Technol..

[17] Fauziyah S.N., Mubarak A.S., Pujiastuti D.Y. (2021). Application of glycerol on bioplastic based carrageenan waste cellulose on biodegradability and mechanical properties bioplastic. IOP Conf. Ser. Earth Environ. Sci..

[18] Vieira M.G.A., da Silva M.A., Dos Santos L.O., Beppu M.M. (2011). Natural-based plasticizers and biopolymer films: A review. Eur. Polym. J..

[19] Amarakoon M., Alenezi H., Homer-Vanniasinkam S., Edirisinghe M. (2022). Environmental Impact of Polymer Fiber Manufacture. Macromol. Mater. Eng..

[20] Norgren M., Costa C., Alves L., Eivazi A., Dahlström C., Svanedal I., Edlund H., Medronho B. (2023). Perspectives on the Lindman Hypothesis and Cellulose Interactions. Molecules.

[21] Kihlman M., Medronho B.F., Romano A.L., Germgård U., Lindman B. (2013). Cellulose Dissolution in an Alkali Based Solvent: Influence of Additives and Pretreatments. J. Braz. Chem. Soc..

[22] Lawson L., Degenstein L.M., Bates B., Chute W., King D., Dolez P.I. (2022). Cellulose Textiles from Hemp Biomass: Opportunities and Challenges. Sustainability.

[23] Zamora-Mendoza L., Guamba E., Miño K., Romero M.P., Levoyer A., Alvarez-Barreto J.F., Machado A., Alexis F. (2022). Antimicrobial Properties of Plant Fibers. Molecules.

[24] Gul R., Jan S.U., Faridullah S., Sherani S., Jahan N. (2017). Preliminary Phytochemical Screening, Quantitative Analysis of Alkaloids, and Antioxidant Activity of Crude Plant Extracts from Ephedra intermedia Indigenous to Balochistan. Sci. World J..

[25] Fukunaga Y., Ogawa R., Homma A., Okada T. (2023). Thin Layer Chromatography-Freeze Surface-Enhanced Raman Spectroscopy: A Powerful Tool for Monitoring Synthetic Reactions. Chem. A Eur. J..

[26] Dar A., Ahmad M.N., Samin G., Jahangir M.M., Rehman R., Anwar J., Al-Thagafi Z.T., Meraf Z., Jaber M.M., Dar A.A. (2023). Separation of Amino Acids, Dyes, and Pigments Using Novel Pressurized Circular TLC Assembly for Secure Medical Imaging Applications. Int. J. Anal. Chem..

[27] Harish D., Solanki N. (2018). Chromatographic methods used for characterization of boswellic acids. MOJ Drug Des. Dev. Ther..

[28] Skalicka-Woźniak K., Garrard I. (2014). Counter-current chromatography for the separation of terpenoids: A comprehensive review with respect to the solvent systems employed. Phytochem. Rev..

[29] Nasser A.L.M., Mazzolin L.P., Hiruma-Lima C.A., Santos L.S., Eberlin M.N., Brito A.R.M.d.S., Vilegas W. (2006). Preparative Droplet Counter-Current Chromatography for the Separation of the New Nor-Seco-Triterpene and Pentacyclic Triterpenoids from Qualea Parviflora. Chromatographia.

[30] Salim M.F.H., Nugraha I.M.A.D.P., Adilla F., Yanti L.P.D. (2021). Chromatography Profiles of Terpenoid Compounds in The Extract of Sambiloto (*Andrographis paniculata*) Herb from Various Solvents. Walisongo J. Chem..

[31] Heftmann E. (2004). Chromatography: Fundamentals and Applications of Chromatography and Related Differential Migration Methods.

[32] Annadurai P. (2021). Extraction and Isolation of bioactive compounds from Lantana camara leaves by column Chromatographic techniques. Res. J. Pharm. Technol..

[33] Yap C.W. (2011). PaDEL-descriptor: An open source software to calculate molecular descriptors and fingerprints. J. Comput. Chem..

[34] Deng C., Deng Y., Wang B., Yang X. (2002). Gas chromatography–mass spectrometry method for determination of phenylalanine and tyrosine in neonatal blood spots. J. Chromatogr. B.

[35] Portella E.H., Romanzini D., Angrizani C.C., Amico S.C., Zattera A.J. (2016). Influence of Stacking Sequence on the Mechanical and Dynamic Mechanical Properties of Cotton/Glass Fiber Reinforced Polyester Composites. Mater. Res..

[36] Hanry E.L., Redzwan N.F.M., Badeges N.F.A.K., Surugau N. (2022). Characterization of biofilms developed from alginate extracted from Padina sp. incorporated with calcium chloride (CaCl_2_). J. Phys. Conf. Ser..

[37] Oh S.Y., Yoo D.I., Shin Y., Kim H.C., Kim H.Y., Chung Y.S., Park W.H., Youk J.H. (2005). Crystalline structure analysis of cellulose treated with sodium hydroxide and carbon dioxide by means of X-ray diffraction and FTIR spectroscopy. Carbohydr. Res..

[38] Krishnamurthy A., Amritkumar P. (2019). Synthesis and characterization of eco-friendly bioplastic from low-cost plant resources. SN Appl. Sci..

[39] Phosri S., Kunjiek T., Mukkhakang C., Suebthep S., Sinsup W., Phornsirigarn S., Charoeythornkhajhornchai P. (2022). Biodegradability of bioplastic blown film in a marine environment. Front. Mar. Sci..

[40] Oliveros R D.A., Machado R.A., Mora J.R. (2022). Quantitative structure–property relationship analysis of the spectrochemical series by employing electronic descriptors from DFT calculations. Mol. Phys..

[41] García-Jacas C.R., Marrero-Ponce Y., Cortés-Guzmán F., Suárez-Lezcano J., Martinez-Rios F.O., García-González L.A., Pupo-Meriño M., Martinez-Mayorga K. (2019). Enhancing Acute Oral Toxicity Predictions by using Consensus Modeling and Algebraic Form-Based 0D-to-2D Molecular Encodes. Chem. Res. Toxicol..

[42] Steven S., Fauza A.N., Mardiyati Y., Santosa S.P., Shoimah S.M. (2022). Facile Preparation of Cellulose Bioplastic from *Cladophora* sp. Algae via Hydrogel Method. Polymers.

[43] Sayakulu N.F., Soloi S. (2022). The Effect of Sodium Hydroxide (NaOH) Concentration on Oil Palm Empty Fruit Bunch (OPEFB) Cellulose Yield. J. Phys. Conf. Ser..

[44] Abubakar A.R., Haque M. (2020). Preparation of Medicinal Plants: Basic Extraction and Fractionation Procedures for Experimental Purposes. J. Pharm. Bioallied Sci..

[45] Nortjie E., Basitere M., Moyo D., Nyamukamba P. (2022). Extraction Methods, Quantitative and Qualitative Phytochemical Screening of Medicinal Plants for Antimicrobial Textiles: A Review. Plants.

[46] Wang Q., Cai J., Zhang L., Xu M., Cheng H., Han C.C., Kuga S., Xiao J., Xiao R. (2013). A bioplastic with high strength constructed from a cellulose hydrogel by changing the aggregated structure. J. Mater. Chem. A.

[47] Kumar S.S., Manoj P., Giridhar P. (2015). Fourier transform infrared spectroscopy (FTIR) analysis, chlorophyll content and antioxidant properties of native and defatted foliage of green leafy vegetables. J. Food Sci. Technol..

[48] Zamora-Mendoza L., Vispo S.N., De Lima L., Mora J.R., Machado A., Alexis F. (2023). Hydrogel for the Controlled Delivery of Bioactive Components from Extracts of *Eupatorium glutinosum* Lam. Leaves. Molecules.

[49] Barthwal R., Mahar R. (2024). Exploring the Significance, Extraction, and Characterization of Plant-Derived Secondary Metabolites in Complex Mixtures. Metabolites.

[50] Kowalska T., Sajewicz M. (2022). Thin-Layer Chromatography (TLC) in the Screening of Botanicals–Its Versatile Potential and Selected Applications. Molecules.

[51] Fathanah U., Lubis M.R., Nasution F., Masyawi M.S. (2018). Characterization of bioplastic based from cassava crisp home industrial waste incorporated with chitosan and liquid smoke. IOP Conf. Ser. Mater. Sci. Eng..

[52] Wang Q., Guo J., Xu D., Cai J., Qiu Y., Ren J., Zhang L. (2015). Facile construction of cellulose/montmorillonite nanocomposite biobased plastics with flame retardant and gas barrier properties. Cellulose.

[53] Shafqat A., Al-Zaqri N., Tahir A., Alsalme A. (2021). Synthesis and characterization of starch based bioplatics using varying plant-based ingredients, plasticizers and natural fillers. Saudi J. Biol. Sci..

[54] Gini T., Jothi G.J. (2018). Column chromatography and HPLC analysis of phenolic compounds in the fractions of *Salvinia molesta* mitchell. Egypt. J. Basic Appl. Sci..

