# Supplementary material for: Bioplastic Production Using Natural Extracts with Cellulose Assisted by Experimental and Computational Screening
Source: Molecules. 2025 Jun 26;30(13):2752. doi: 10.3390/molecules30132752 (PMC12251182; doi:10.3390/molecules30132752)
Supplement: Supplementary file 1 [file molecules-30-02752-s001.zip › molecules-3638756-supplementary.pdf]

Supplementary Materials:

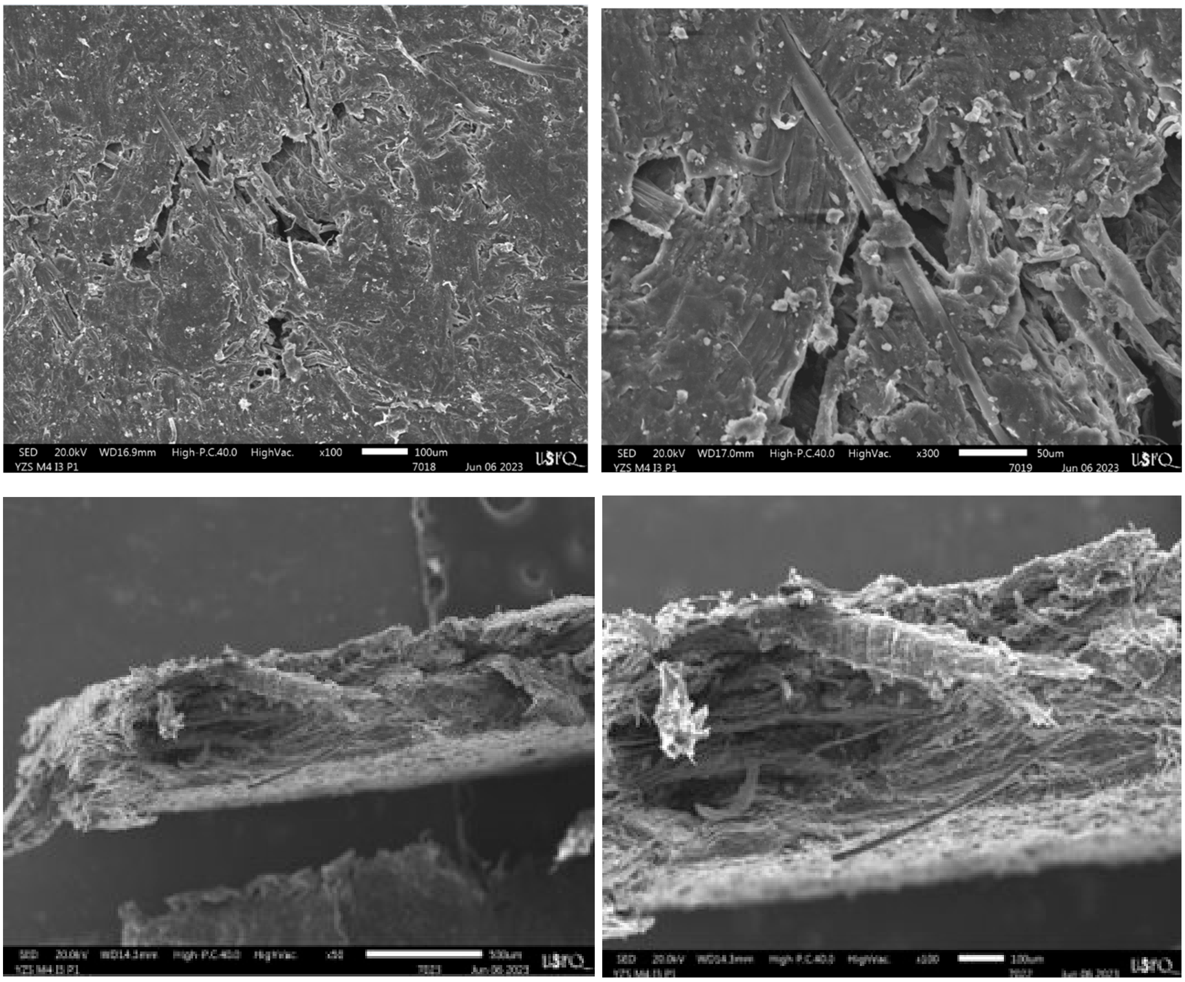

a) D1 W

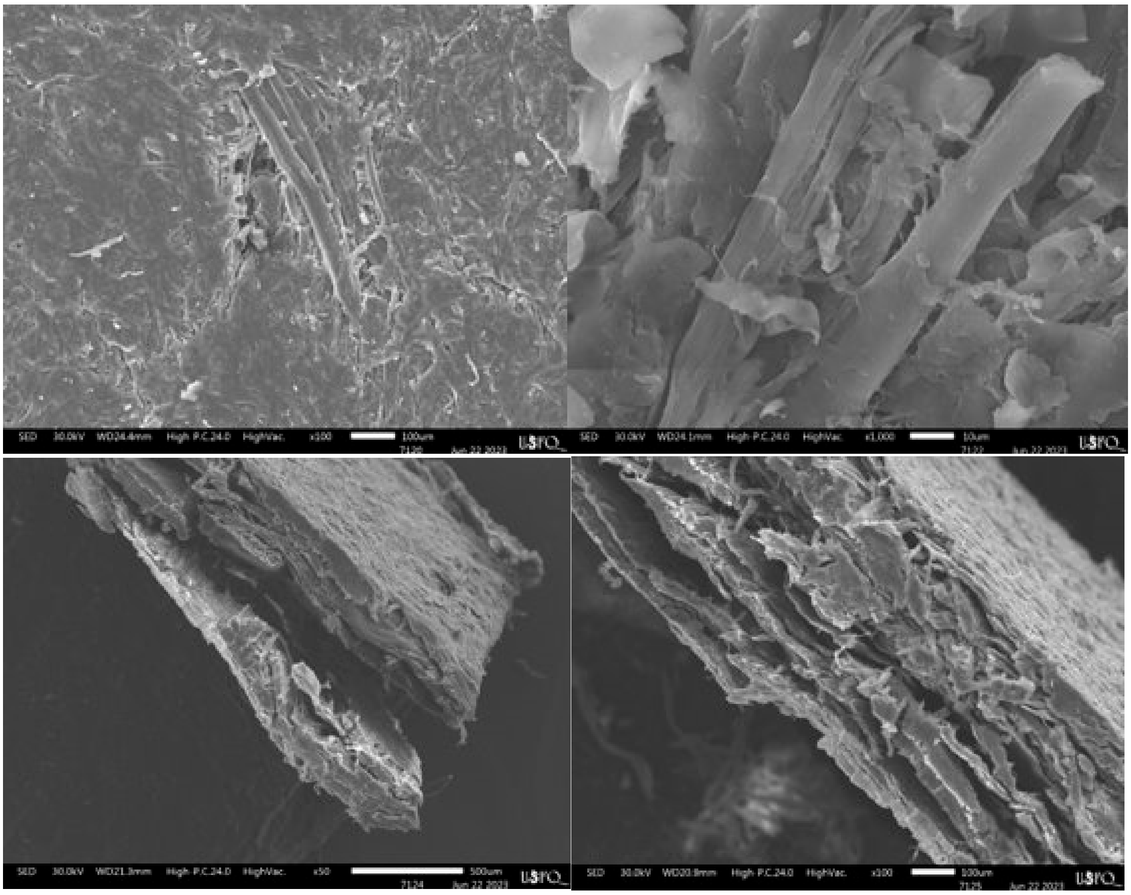
SED 30.0kV WD24.4mm High P.C.24.0 HighVac. x100 100um
SED 30.0kV WD24.1mm High P.C.24.0 HighVac. x1,000 10um
SED 30.0kV WD21.3mm High P.C.24.0 HighVac. x50 500um
SED 30.0kV WD20.8mm High P.C.24.0 HighVac. x100 100um

b) D2 W

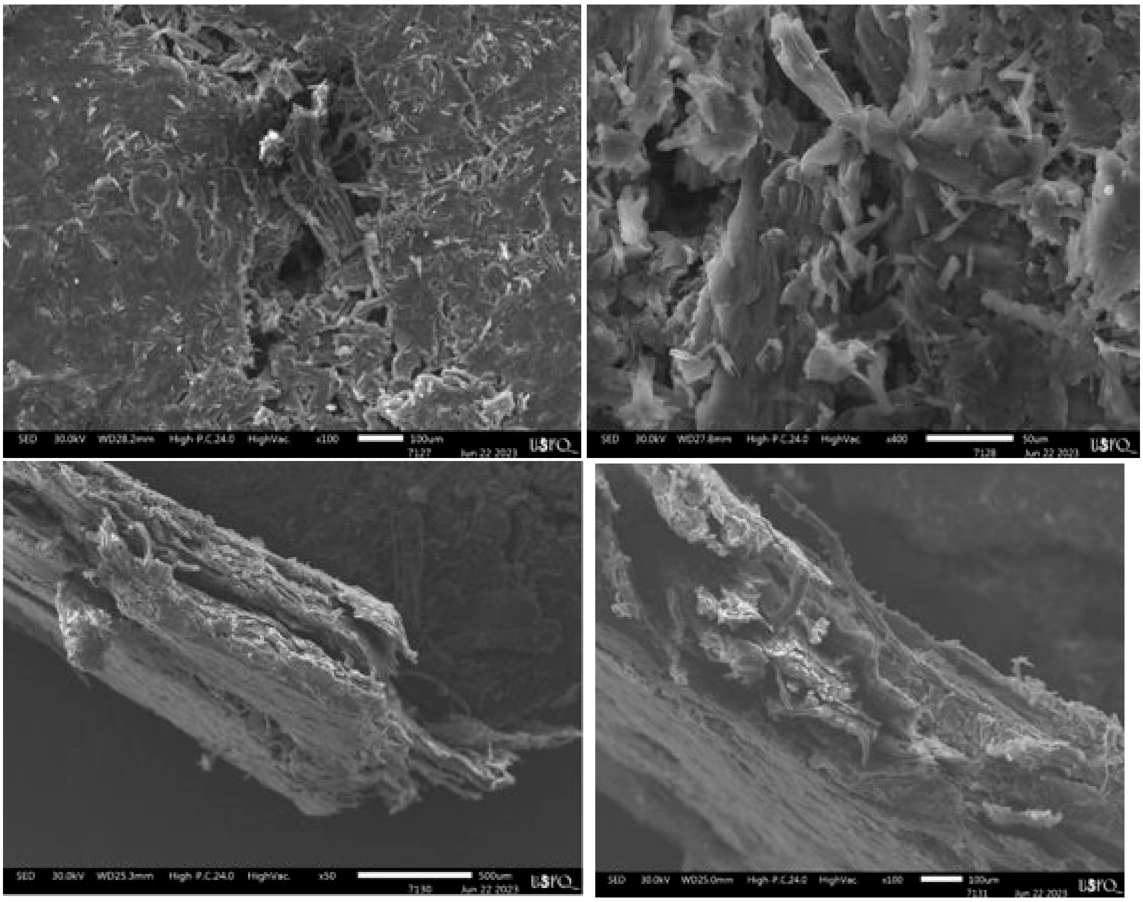

c) D3 W

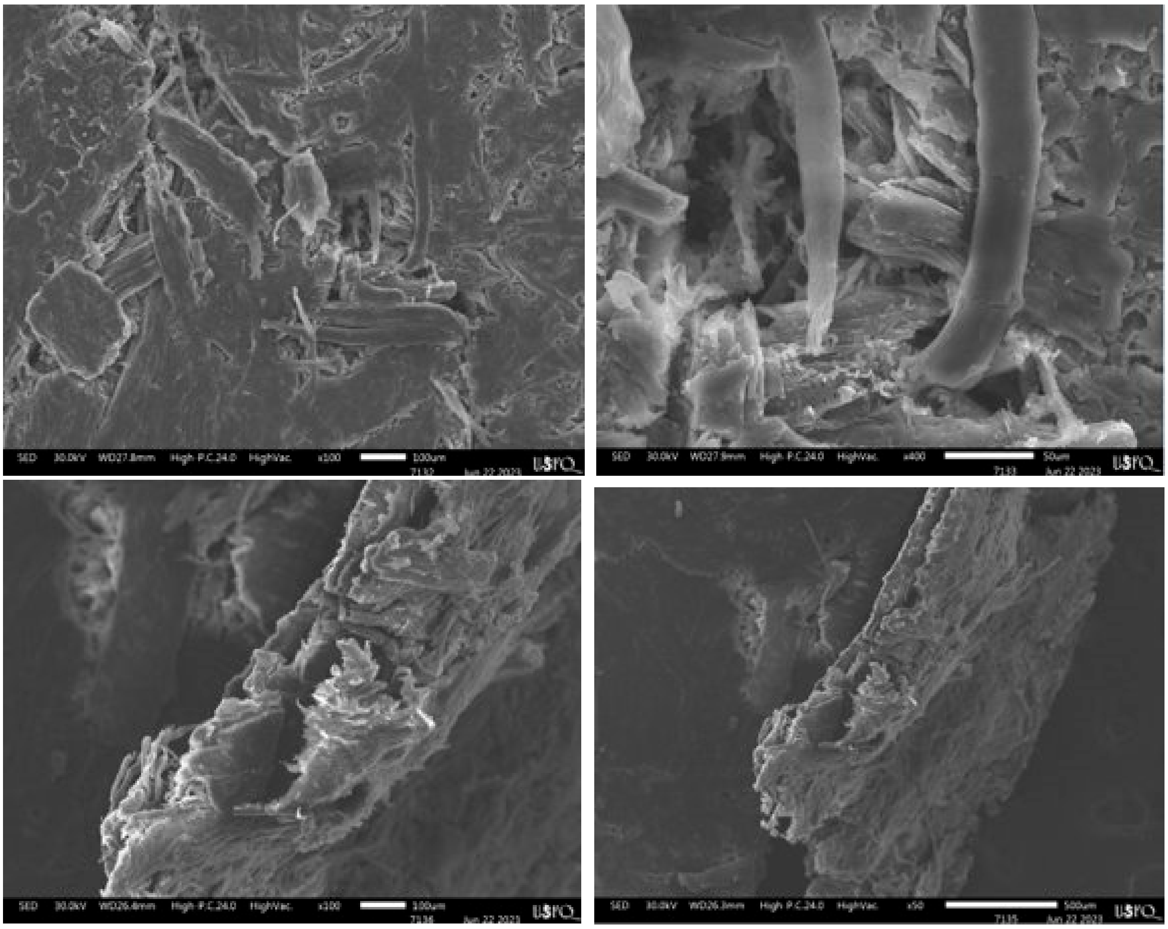

d) D4 W

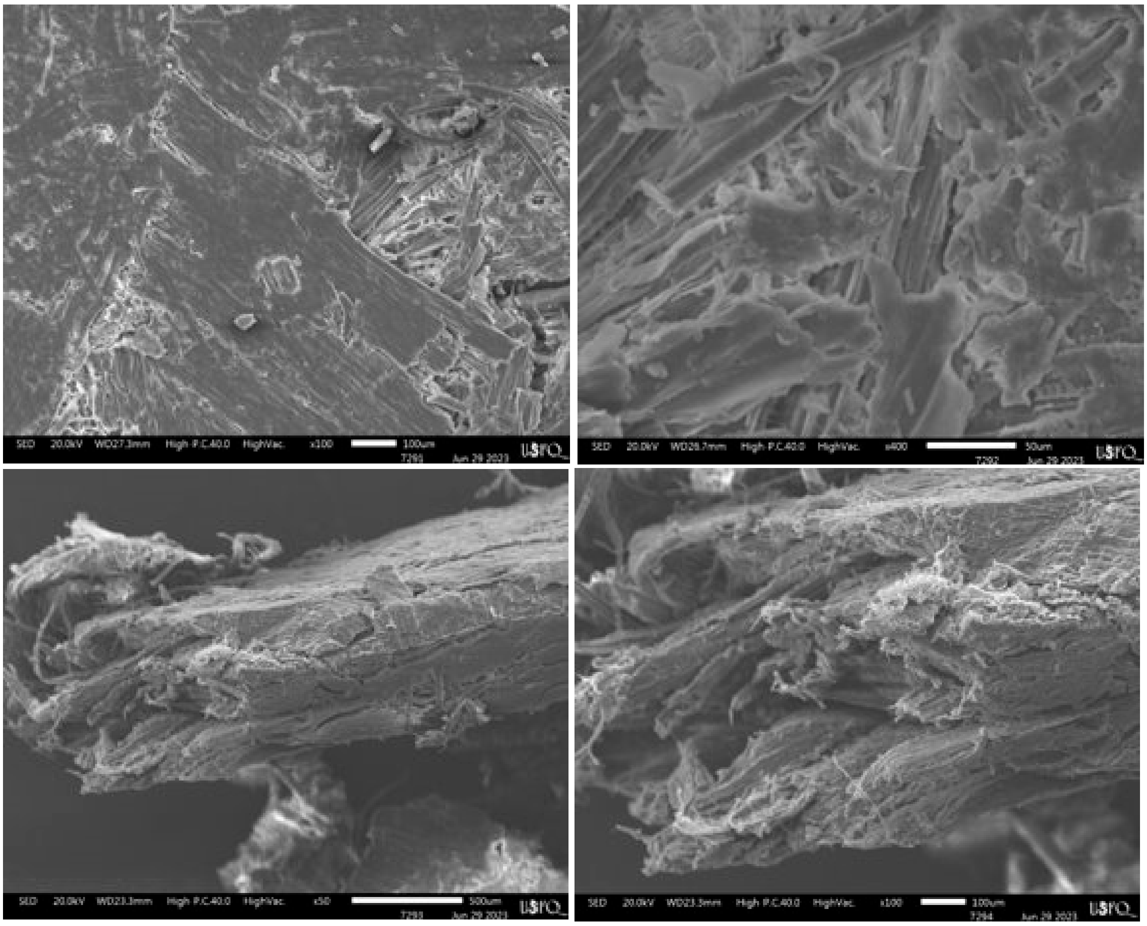

e) D5 W

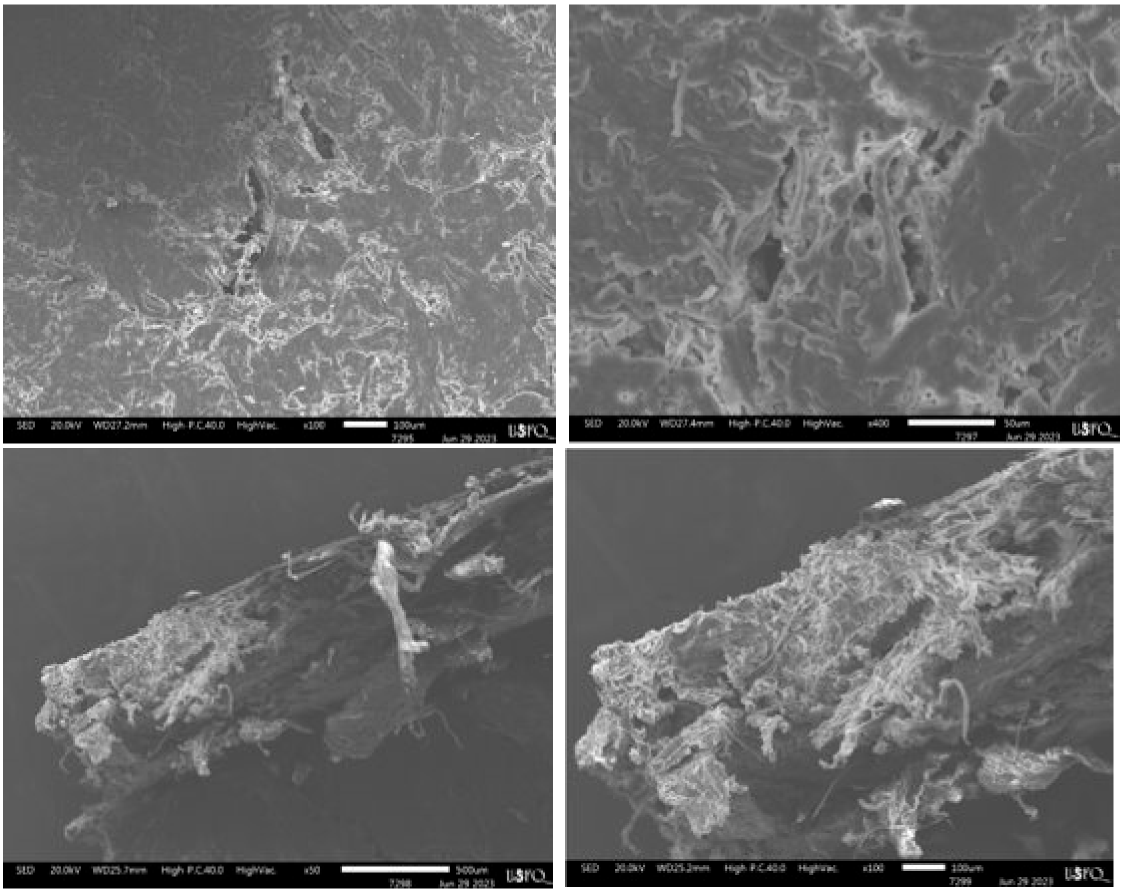

f) D6 W

Figure S1. SEM images of the bioplastic films of quinoa (D1 W), guayaba (D2 W), palo santo (D3 W), Ivory Coast almond (D4 W), cacalosuchil (D5 W), and soya (D6 W) at the highest concentration of 15 wt.% at different magnifications.

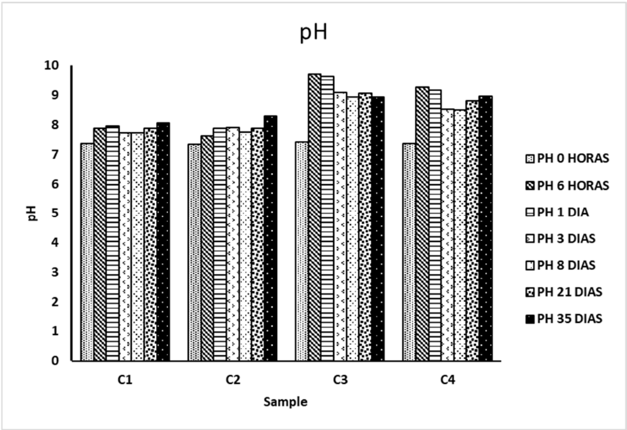

a) Controls

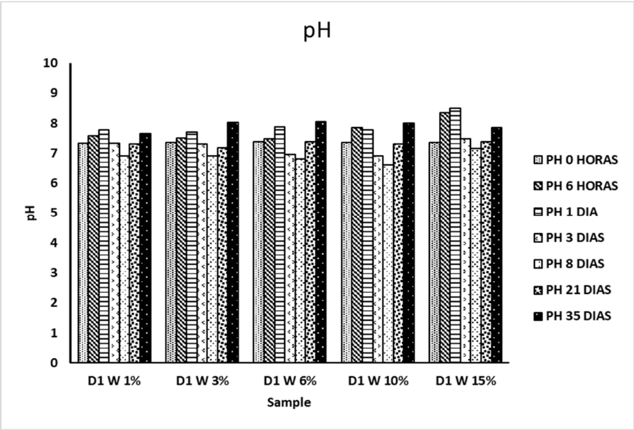

b) D1 W

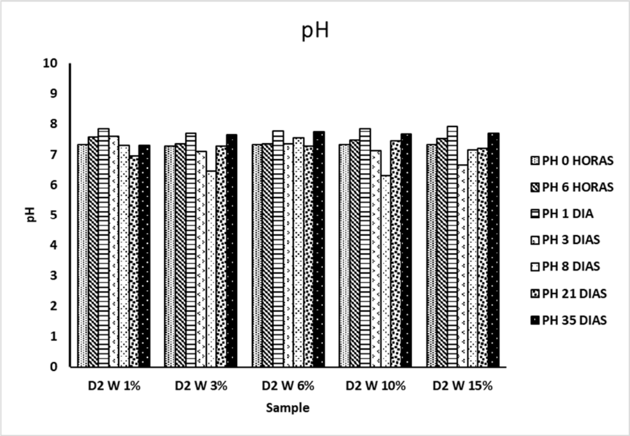

c) D2 W

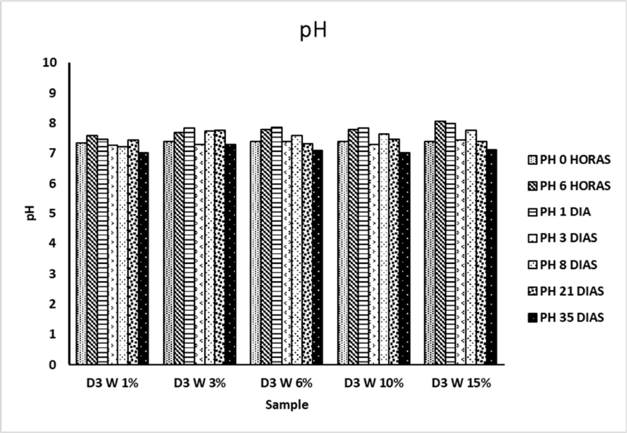

d) D3 W

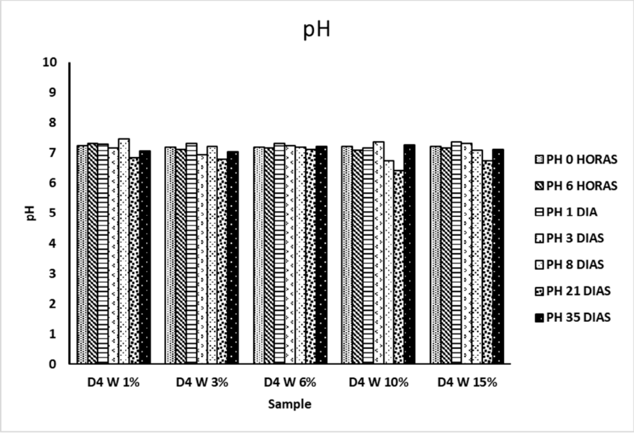

e) D4 W

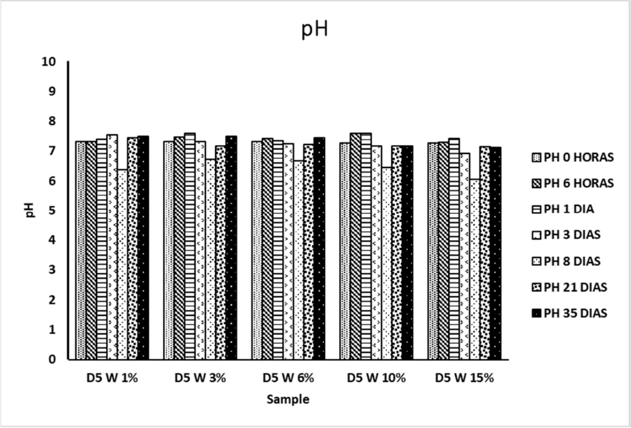

f) D5 W

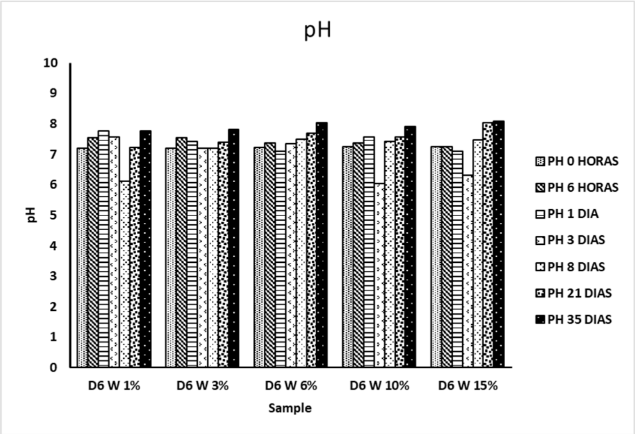

g) D6 W

Figure S2. pH change in degradation in water test at 0 h, 6 h, 1 day, 3 days, 8 days, 21 days, and 35 days of the bioplastics films with plant extract and conventional plasticizers.
